# Ubr3, a Novel Modulator of Hh Signaling Affects the Degradation of Costal-2 and Kif7 through Poly-ubiquitination

**DOI:** 10.1371/journal.pgen.1006054

**Published:** 2016-05-19

**Authors:** Tongchao Li, Junkai Fan, Bernardo Blanco-Sánchez, Nikolaos Giagtzoglou, Guang Lin, Shinya Yamamoto, Manish Jaiswal, Kuchuan Chen, Jie Zhang, Wei Wei, Michael T. Lewis, Andrew K. Groves, Monte Westerfield, Jianhang Jia, Hugo J. Bellen

**Affiliations:** 1 Program in Developmental Biology, Baylor College of Medicine, Houston, Texas, United States of America; 2 Markey Cancer Center and Department of Molecular and Cellular Biochemistry, University of Kentucky, Lexington, Kentucky, United States of America; 3 Institute of Neuroscience, University of Oregon, Eugene, Oregon, United States of America; 4 Department of Molecular and Human Genetics, Baylor College of Medicine, Houston, Texas, United States of America; 5 Jan and Dan Duncan Neurological Research Institute, Texas Children’s Hospital, Houston, Texas, United States of America; 6 Howard Hughes Medical Institute, Baylor College of Medicine, Houston, Texas, United States of America; 7 Department of Molecular and Cellular Biology, Baylor College of Medicine, Houston, Texas, United States of America; 8 Lester and Sue Smith Breast Center, Baylor College of Medicine, Houston, Texas, United States of America; 9 Department of Neuroscience, Baylor College of Medicine, Houston, Texas, United States of America; New York University, UNITED STATES

## Abstract

Hedgehog (Hh) signaling regulates multiple aspects of metazoan development and tissue homeostasis, and is constitutively active in numerous cancers. We identified Ubr3, an E3 ubiquitin ligase, as a novel, positive regulator of Hh signaling in *Drosophila* and vertebrates. Hh signaling regulates the Ubr3-mediated poly-ubiquitination and degradation of Cos2, a central component of Hh signaling. In developing *Drosophila* eye discs, loss of *ubr3* leads to a delayed differentiation of photoreceptors and a reduction in Hh signaling. In zebrafish, loss of Ubr3 causes a decrease in Shh signaling in the developing eyes, somites, and sensory neurons. However, not all tissues that require Hh signaling are affected in zebrafish. Mouse UBR3 poly-ubiquitinates Kif7, the mammalian homologue of Cos2. Finally, loss of UBR3 up-regulates Kif7 protein levels and decreases Hh signaling in cultured cells. In summary, our work identifies Ubr3 as a novel, evolutionarily conserved modulator of Hh signaling that boosts Hh in some tissues.

## Author Summary

Hedgehog signaling regulates many important biological processes and has been linked to developmental disorders, wound healing, and cancer. Although the major components in the pathway have been well studied in *Drosophila* and vertebrates, how the signaling is regulated by different modulators is not well understood. Here, we take advantage of a fly forward genetic screen to isolate Ubr3. We show that it is a novel modulator that regulates Hh signaling. Loss of *ubr3* leads to Hh signaling defects in developing eyes of *Drosophila*, and affects eye, and somite and sensory neuron development in zebrafish. However, Hh signaling is not affected in all cells known to be dependent on Hh signaling as loss of *ubr3* in the fly wing and zebrafish inner ear are not affected. This suggests that Ubr3 is a modulator that is only required in some Hh dependent organs/cells. We have shown that Ubr3 down-regulates the levels of Cos2 and its mammalian homolog Kif7, key negative regulators of Hh signaling, through poly-ubiquitination. The poly-ubiquitination of Cos2 by Ubr3 is enhanced by Hh activation, suggesting that it functions in a positive feedback that modulates Hh activation.

## Introduction

**Fig 1:**
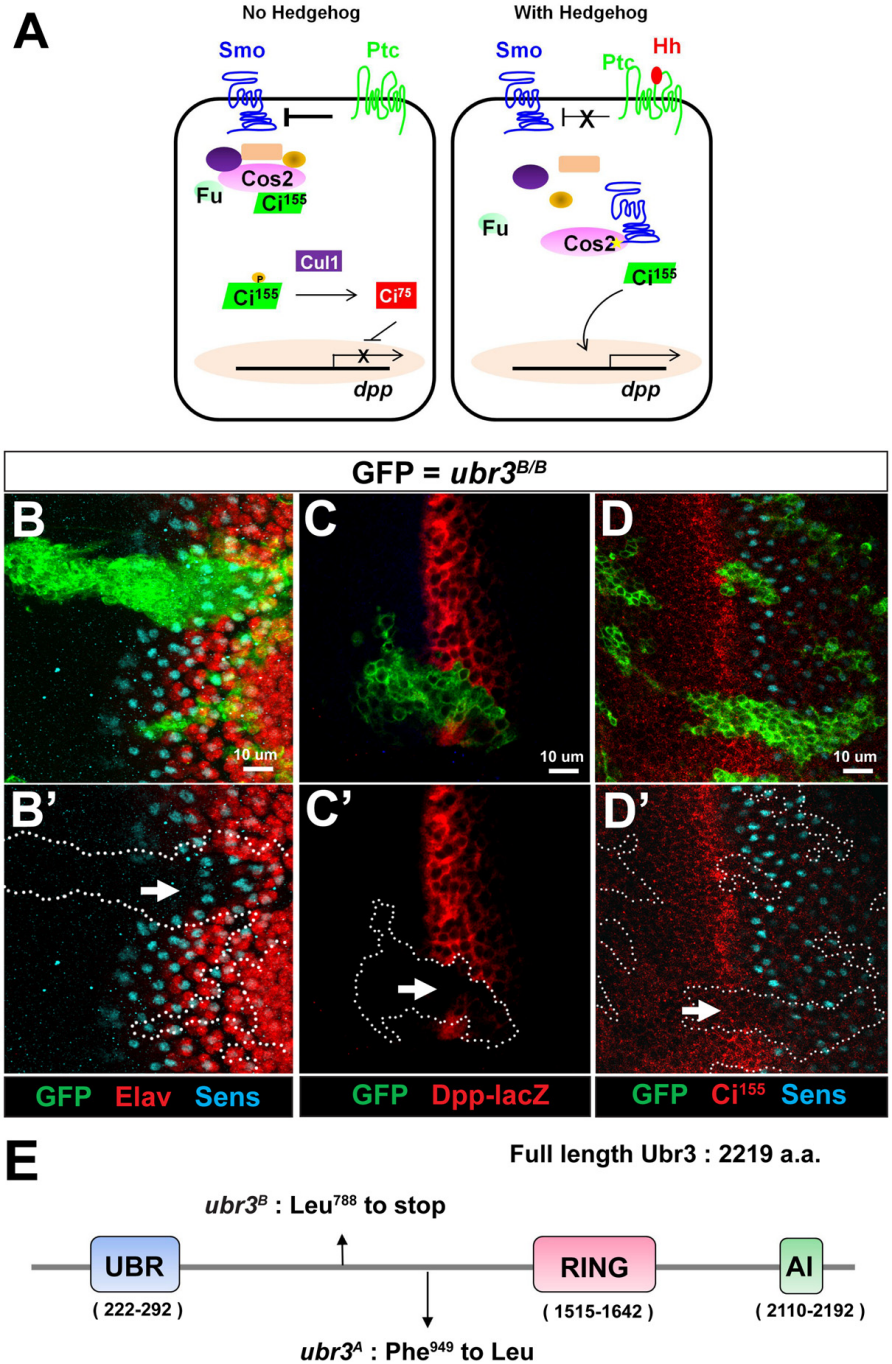
Loss of *ubr3* leads to Hh signaling defects in the morphogenetic furrow of eye imaginal discs. (A) Simplified diagram of Hh signaling transduction pathway in *Drosophila*. (B-B’) *ubr3*^*B*^ mutant clones (labeled by GFP) crossing the morphogenetic furrow exhibit delayed differentiation of photoreceptors (arrow), revealed by expression of Sens (cyan) and Elav (red). (C-C’) Expression of *dpp-lacZ* (red) is lost (arrow) in *ubr3*^*B*^ mutant cells (labeled by GFP). (D-D’) *ubr3*^*B*^ mutant clones (labeled by GFP) exhibit loss of Ci^155^ labeling (red) in the morphogenetic furrow (arrow) and delay of R8 photoreceptor differentiation, visualized by the delayed expression of Sens (cyan). All images of eye discs are positioned with their anterior side to the left. (E) Schematic diagram shows the conserved domains of the Ubr3 protein and the molecular lesions (Leu^788^>STOP and Phe^949^>Leu) identified in the *ubr3*^*B*^ and *ubr3*^*A*^ alleles respectively.

Hedgehog (Hh) signaling regulates numerous developmental processes and is implicated in multiple cancers, wound healing and pain sensation in adults [1–3]. The Hh ligand acts as a morphogen to induce differential cell responses based on distinct activity thresholds of its signaling transduction cascade [4–6]. Mis-regulation of Hh signaling affects cell specification and proliferation during development and causes several types of cancer such as glioblastoma or basal cell carcinoma [7, 8]. In the absence of Hh, the receptor Patched (Ptc) inhibits the G-protein coupled receptor Smoothened (Smo) [9]. Inhibition of Smo promotes the assembly of an antagonistic molecular complex composed of Costal 2 (Cos2), a kinesin-related motor protein, Cubitus interruptus (Ci), the key transcriptional effector of Hh [10, 11], and several protein kinases [12]. This complex phosphorylates the full length, transcriptionally active form of Ci, Ci^155^. Phosphorylated Ci^155^ is ubiquitinated by a SCF (Skp1-Cullin1(Cul1)-F-box) E3 ligase complex [13] and partially cleaved to generate a transcriptional repressor form, Ci^75^, which leads to the transcriptional silencing of Hh target genes [14, 15]. The Hh signaling cascade is activated by the binding of Hh to Ptc and Ihog (Interference hedgehog) [16], resulting in the release of Smo inhibition. Activated Smo can interact physically with Cos2 [17–20]. This interaction prevents the formation of the Hh signaling antagonistic complex and cleavage of Ci^155^. As a result, levels of Ci^155^ increase in the cytoplasm, promoting its translocation to the nucleus and the transcription of downstream target genes such as *decapentaplegic* (*dpp*) or *ptc* (Fig 1A).

Previous studies have shown that Cos2 is a key modulator of Hh signaling, and that it facilitates kinase-mediated phosphorylation of Ci and promotes partial degradation of Ci [21]. Loss of Cos2 leads to ectopic activation of Hh signaling and pattern duplications in the *Drosophila* wing [11], whereas over-expression of Cos2 inhibits Hh signaling [22], suggesting that Cos2 is both necessary and sufficient for Hh signaling. In vertebrates, the core components of Hh signaling are conserved, including Cos2. Cos2 has two vertebrate orthologs, Kif7 and Kif27 [23, 24]. Kif7 has been proposed to function similarly to Cos2, because *Kif7* knockout mice and zebrafish mutants show an up-regulation of Sonic Hedgehog (Shh) signaling [25–27]. In addition, Kif7 can interact physically and modulate the activity of the GLI transcription factors, the mammalian homologs of Ci [27, 28]. Moreover, Cos2 can functionally replace Kif7 [27], demonstrating a molecular conservation between vertebrate and invertebrate homologues. In humans, patients carrying *KIF7* allelic variants display a spectrum of phenotypic severity ranging from hydrolethalus or Acrocallosal syndromes to Meckel and Joubert syndromes [28, 29]. Hence, proper function of Kif7 activity is essential for correct Hh signal transduction and is likely to be regulated tightly. Previous studies have shown that Cos2 (Kif7) is phosphorylated by a kinase, Fused, which mediates the strength of differential Hedgehog signaling [30, 31]. To date, however, no data support a role for ubiquitination in the regulation of Cos2.

Ubiquitination plays an important role in several steps of Hh signaling [32–34]. Ubiquitination is catalyzed by a cascade of enzymes consisting of ubiquitin-activating (E1), -conjugating (E2), and –ligating (E3) enzymes [35]. E3 enzymes bind, transfer and ligate ubiquitin to particular substrates. The two major types of E3 ligase are the Really Interesting New Gene (RING) domain E3s and the Homologous to E6AP Carboxyl Terminus (HECT) domain E3s [36].

We describe the identification and characterization of Ubr3, a novel regulator of Hh signaling. Ubr3 belongs to the UBR protein superfamily, characterized by a 70-residue zinc finger domain UBR box [37]. Recent studies showed that Ubr3 can polyubiquitinate target proteins [38] involved in multiple biological processes, including olfactory organ function in mice [39], denticle patterning in *Drosophila* [40], DNA damage repair in yeast [38], apoptosis in flies [41], homoeostasis in the heart [42], and breast cancer [43].

Here we show that Ubr3 promotes Hh signaling by mediating the ubiquitination and degradation of Cos2/Kif7. Loss of Ubr3 elevates the levels of Cos2, resulting in a decrease in Ci^155^ and transcriptional silencing of Hh target genes. Loss of *ubr3* in flies and zebrafish affects eye development, as well as neuronal specification and somite development in zebrafish. Ubr3 regulates the ubiquitination and degradation of Kif7 in mammalian cells, and transcription of the Shh target *ptch2* is strongly decreased in the retina of *ubr3* mutant zebrafish. Taken together, our data suggest that Ubr3 is an evolutionarily conserved, positive regulator of Hh signaling that regulates Cos2/Kif7 ubiquitination and degradation.

## Results

### Loss of *ubr3* results in Hh signaling defects in *Drosophila*

To identify novel components in developmental signaling pathways, we isolated mutations that affect eye and/or wing morphogenesis in a mosaic forward genetic screen of approximately 6000 X-linked lethal mutations in *Drosophila* [44–48]. We identified an essential complementation group *ubr3*, consisting of two alleles (*ubr3*^*A*^ and *ubr3*^*B*^). Both *ubr3*^*A*^ and *ubr3*^*B*^ hemizygous mutants die as 1^st^ instar larvae. Homozygous mutant clones of both alleles cause delayed differentiation of photoreceptors in the morphogenetic furrow of eye imaginal discs (Fig 1B and S1A Fig). This is revealed by the delayed expression of Senseless, an R8 photoreceptor marker [49, 50] and Elav (Embryonic lethal abnormal vision), a marker for photoreceptors [51]. Since delayed differentiation of photoreceptors is observed when Hh signaling is lost [52], we hypothesized that *ubr3* mutations may impair Hh signaling. To assess the activation of Hh signaling in *ubr3* mutant clones, we examined expression of a Hh reporter, *dpp-lacZ* [53] and the active form of Ci, Ci^A^. Both *dpp-lacZ* and Ci^155^ are lost in *ubr3* mutant clones in the morphogenetic furrow (Fig 1C–1D’ and S1B Fig). We and others also noticed an increase in apoptosis in *ubr3* mutant cells [41]. To exclude the possibility that the Hh signaling defect in *ubr3* mutant cells is due to apoptosis, we over-expressed the anti-apoptotic gene *p35* in *ubr3* clones. As shown in S1C Fig, the delayed differentiation of photoreceptors is not rescued although apoptosis is suppressed (S1D and S1E Fig). Hence, Hh signaling is impaired in *ubr3* mutant cells.

Alleles of *ubr3* map to a small deficiency that uncovers ~11 genes including *ubr3* (*CG42593*) and *l(1)G0193* (S1F Fig). Both alleles (*ubr3*^*A*^ and *ubr3*^*B*^) fail to complement the lethality associated with a *P-*element insertion in *ubr3* (S1F and S1H Fig) [54]. *ubr3*^*B*^ carries a Leu^788^>STOP and *ubr3*^*A*^ carries a Phe^949^>Leu in *ubr3* (Fig 1E). No mutations were found in *l(1)G0193*. A genomic rescue construct rescued the lethality of both *ubr3* alleles (S1F and S1H Fig), and over-expression of the *ubr3* cDNA in *ubr3*^*B*^ mutant clones rescued the loss of Ci^155^ expression in the morphogenetic furrow (S1G Fig). Together, these data show that *ubr3* is required for Hh signaling.

### Ubr3 is a conserved E3 ligase and is expressed in *Drosophila* eye discs

*ubr3* encodes a 2219 amino acid protein, the *Drosophila* homolog of the mammalian RING-type E3 ubiquitin ligase n-recognin 3 (*UBR3*) gene (Fig 1E). Most UBR superfamily member proteins, including UBR1, UBR2, UBR4 and UBR5, function in the N-end rule pathway, a ubiquitin-dependent system where E3 ligases recognize N terminal residues of their targets and degrade them [37]. However, UBR3 does not bind to known N-end rule substrates, suggesting a different molecular function of Ubr3 from N-end rule E3 ligases [55]. Ubr3 contains a UBR moiety, a RING domain and a C-terminal auto-inhibitory (AI) domain (Fig 1E) [38, 39]. All three domains are highly conserved among fly, mouse and human (S1I Fig), suggesting that the molecular function of Ubr3 may be conserved.

**Fig 2:**
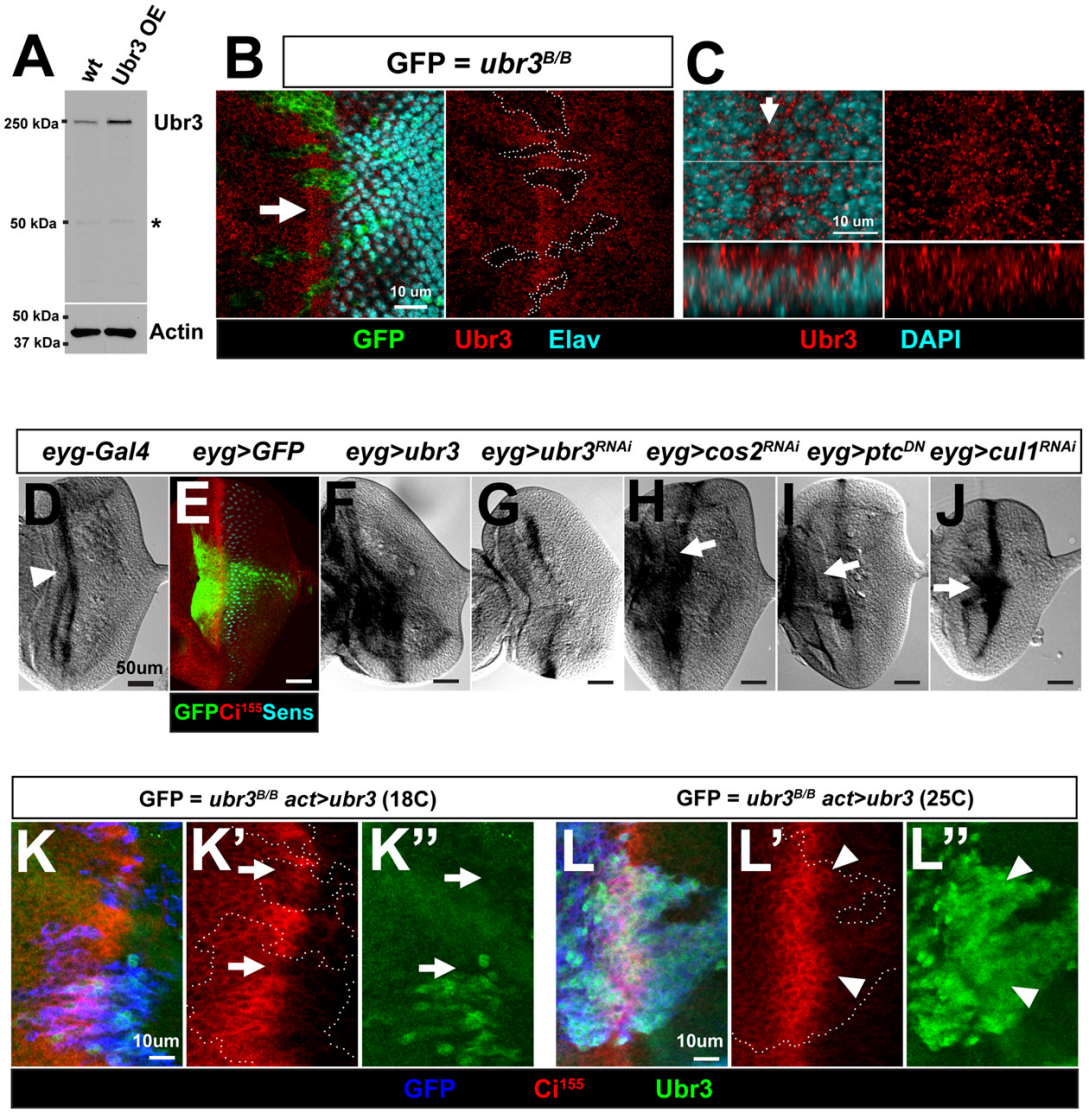
The transcription of *ubr3* is up-regulated in the morphogenetic furrow by Hh signaling activation, forming a positive feedback regulatory loop. (A) Western blot analysis of wild-type (wt) larval eye-brain lysates from *y w* flies or flies over-expressing *ubr3* under the control of *GMR-Gal4* (*GMR-Gal4>UAS-Ubr3)*, probed with the anti-Ubr3 polyclonal antibody (upper panel) reveals a band of the expected size of 250kDa (arrowhead), corresponding to endogenous or over-expressed Ubr3 protein. The asterisk indicates a non-specific band recognized by GP-Ubr3. (B) Anti-Ubr3 antibody labeling of eye discs containing *ubr3*^*B*^ mutant clones (labeled with GFP) document the specificity of the antibody, and reveals the broad expression of Ubr3 protein. Ubr3 is enriched in the morphogenetic furrow (arrow). Elav (cyan) marks the photoreceptor cells. (C) Co-immunolabeling of GP-Ubr3 (red) and DAPI (cyan) in the eye disc from third instar larvae indicate that Ubr3 protein (red) localizes to the cytosol and is excluded from nuclei, marked by DAPI (cyan). An XZ confocal section of a region close to the morphogenetic furrow (arrow) is shown, with the anterior side positioned to the left. (D, F-J) In situ hybridization images of eye discs with probes recognizing *ubr3* cDNA. (D) *ubr3* is actively transcribed in the morphogenetic furrow region (arrowhead) in a wild-type (*eyg-Gal4/+*) eye disc. (E) Expression pattern of *eyg-Gal4* driver is shown by a GFP reporter. Ci^155^ (red) marks the morphogenetic furrow. (F-G) *eyg-Gal4* driven over-expression of *ubr3* cDNA and *ubr3* RNAi cause increased and decreased signals respectively in the over-expressed regions of eye discs, suggesting the efficiency and specificity of *ubr3* probes. (H-J) Ectopic activation of Hh signaling (arrows in H to J) in Eyg-Gal4 expressing region (shown in E) through *cos2*^*RNAi*^, *ptc*^*DN*^ or *cul1*^*RNAi*^ induces higher level of *ubr3* transcription (arrows). (K, L) *ubr3* cDNA expression (shown in green) was driven by actin-Gal4 in *ubr3*^*B/B*^ clones cells (labeled in blue in K and L or dashed lines in K’ and L’) at 18°C or 25°C. Ci^155^ labeling is shown in red. Low levels of Ubr3 expression at 18°C cannot fully rescue Ci^155^ expression in *ubr3*^*B/B*^ mutant clones (arrows), while high levels of expression of Ubr3 at 25°C lead to increased expression of Ci^155^ posterior to the morphogenetic furrow (arrowheads).

To determine the expression pattern and protein localization of Ubr3, we raised a polyclonal antibody against a region between UBR domain and RING domain of Ubr3 (see Materials and Methods). The Ubr3 antibody specifically recognized a single 250 kDa band on Western blots of protein extracts from larval eye-brain complexes (Fig 2A). This band became more intense when a Ubr3 transgene was expressed (Fig 2A). Furthermore, immunofluorescent labeling of eye imaginal discs with our Ubr3 antibody revealed that the signal was severely diminished or lost within *ubr3*^*B*^ mutant clones (Fig 2B). Ubr3 is cytosolic and broadly expressed (Fig 2C) and is enriched in the morphogenetic furrow of developing eye discs (Fig 2B), where Ci^155^ and *dpp-lacZ* expression is elevated. The Ubr3 proteins in the cytosol are present in puncta that do not show obvious co-localization with a markers for different organelles (S2A–S2G Fig). These data suggest that elevated levels of Ubr3 positively correlate with the activation of Hh signaling.

### Hh signaling activates the transcription of *ubr3*

To assess whether the enriched Ubr3 protein in the morphogenetic furrow (Fig 2B) results from increased transcription of *ubr3*, we performed *in situ* hybridization experiments. As shown in Fig 2D, *ubr3* was transcribed most abundantly in the morphogenetic furrow, in agreement with the protein enrichment shown in Fig 2B. Over-expression of *ubr3* with an *eyegone-Gal4* driver (*eyg-Gal4*; Fig 2E) expanded *ubr3* expression domain in eye discs (Fig 2F), whereas *ubr3* RNAi knockdown decreased expression of *ubr3* in the center of the eye disc (Fig 2G), showing the specificity of the RNA probes. We activated Hh signaling in the *eyg* positive cells by expressing a dominant-negative Ptc (*ptc*^*DN*^) [56] or by down-regulating the expression of negative Hh regulators Cos2 or Cul1 by RNAi. In all cases, activation of Hh signaling elevated *ubr3* mRNA levels in eye discs (Fig 2H–2J). In contrast, down-regulation of Ci by expressing Ci^RNAi^ in the equator region of the morphogenetic furrow through *eyg-Gal4* (arrow in S2H Fig) resulted in moderate loss of *ubr3* transcription (white arrow in S2I Fig). Hence, Hh signaling positively regulates *ubr3* expression at both the mRNA and protein levels. To assess whether different levels of Ubr3 proteins contribute in a dosage-dependent manner to Hh signaling, we manipulated the expression levels of Ubr3 in *ubr3*^*B/B*^ mutant cells by expressing a *ubr3* cDNA at low or high levels. The *actin-Gal4* driver used to express the *ubr3* cDNA is temperature sensitive and leads to low expression at 18°C and medium to high expression at 25°C [57]. We then assessed Ci^155^ expression in the mutant clones expressing discrete levels of Ubr3. Interestingly, when Ubr3 was expressed at low level at 18°C, Ci^155^ expression was only partially restored (arrows in Fig 2K). However, high level of Ubr3 expression in *ubr3* mutant cells fully rescued Ci^155^ expression. In some cells, Ubr3 over-expression induced ectopic expression of Ci^155^ posterior to the morphogenetic furrow (arrowheads in Fig 2L–2L”). In summary, these data suggest that Hh activation up-regulates transcription of *ubr3*, which in turn promotes Hh signaling.

### Cos2 is up-regulated in *ubr3* mutant cells

**Fig 3:**
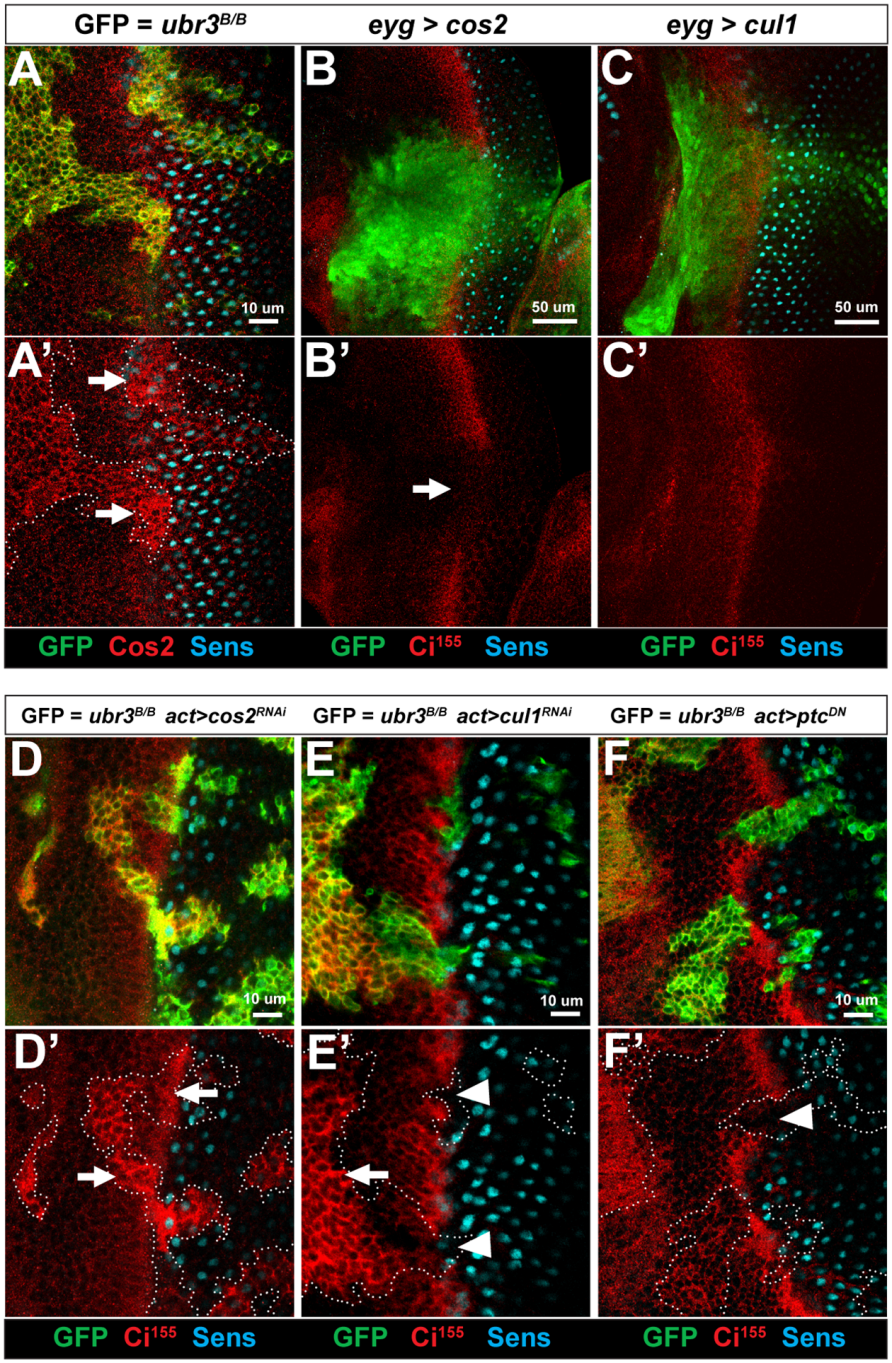
Up-regulation of Cos2 in *ubr3* mutant clones is responsible for the loss of Hh signaling. (A-A’) *ubr3*^*B*^ mutant cells (marked by GFP) up-regulate Cos2 (red; arrows). (B-B’) Over-expression of Cos2 by *eyg-Gal4* (indicated by expression of GFP) leads to loss of Ci^155^ (arrow in B’) at the morphogenetic furrow. (C-C’) Over-expression of Cul1 by *eyg-Gal4* (indicated by expression of GFP) does not cause loss of Ci^155^ at the morphogenetic furrow. (D-D’) Down-regulation of Cos2 by over-expression of *cos2*^*RNAi*^ in *ubr3*^*B*^ mutant clones (marked by the expression of GFP) suppresses loss of Ci^155^ (red) in the morphogenetic furrow (arrows in D’). (E-E’) Down-regulation of Cul1 by over-expression of *cul1*^*RNAi*^ in *ubr3*^*B*^ mutant clones induces ectopic activation of Hh signaling anterior to the morphogenetic furrow (shown by expression of Ci^155^, arrow) but does not rescue Ci^155^ loss in the morphogenetic furrow (arrowheads). (F-F’) Over-expression of *ptc*^*DN*^ in *ubr3*^*B*^ mutant clones does not rescue Ci^155^ loss in the morphogenetic furrow (arrowhead).

To determine how Ubr3 promotes Hh signaling, we assessed the protein expression of key components of the Hh pathway in *ubr3* mutant clones. Expression of Ptc and Fused (Fu), a kinase interacting with Cos2, was not obviously affected (S3A–S3B’ Fig), but Cos2 (Fig 3A and 3A’) and Cul1 (S3C and S3C’ Fig) were up-regulated in *ubr3* mutant eye clones. Cos2 up-regulation is obvious in the morphogenetic furrow (arrows in Fig 3A’), suggesting that Hh regulates the Ubr3-mediated down-regulation of Cos2.

Cos2 and Cul1 are both negative regulators of Hh signaling and loss of function of either gene causes ectopic activation of Hh signaling in eye discs [11, 13, 58]. Because both genes are up-regulated in cells lacking Ubr3, we tested whether over-expression of either gene is sufficient to phenocopy the *ubr3* mutation. Over-expression of Cos2, but not Cul1, results in loss of Ci^155^ in the morphogenetic furrow, similar to *ubr3* mutants (Fig 3B–3C’). Labeling with a Cos2 antibody showed that a subtle increase of Cos2 is sufficient to inhibit Ci^155^ expression (S3D and S3D’ Fig), implicating that Cos2 up-regulation in *ubr3* mutant cells is relevant. Hence, up-regulation of Cos2, but not Cul1, is likely to be responsible for the Hh signaling defects observed in *ubr3* mutants. This hypothesis is supported by the observation that reducing Cos2 protein levels in *ubr3* mutant clones through *cos2*^*RNAi*^ restored Ci^155^ levels and suppressed the morphogenetic furrow defects (arrows in Fig 3D and 3D’ and S3E and S3E’ Fig). In contrast, over-expression of *cul1*^*RNAi*^ in *ubr3* mutant clones did not restore Ci^155^ expression in the morphogenetic furrow (arrowheads in Fig 3E and 3E’), suggesting that Cul1 up-regulation was not the cause of Ci^155^ loss. One likely reason why Ci^155^ expression is not restored by Cul1 RNAi in *ubr3* mutant clones in the morphogenetic furrow is that Cul1 RNAi does not completely remove Cul1 in *ubr3* clones and the residual Cul1-Slimb E3 ligase activity may suffice to mediate processing of Ci^155^. Moreover, expression of *ptc*^*DN*^ in *ubr3* mutant clones did not rescue Ci^155^ loss (arrowhead in Fig 3F and 3F’). These data show that loss of *ubr3* causes a decrease in Hh signaling and a reduction in Ci^155^ that can be restored by Cos2 down-regulation. Hence, *ubr3* acts to attenuate the levels of Cos2, which enhances the activity of Hh signaling in the morphogenetic furrow.

### Ubr3 possesses Ubiquitin E3 ligase activity and ubiquitinates itself

**Fig 4:**
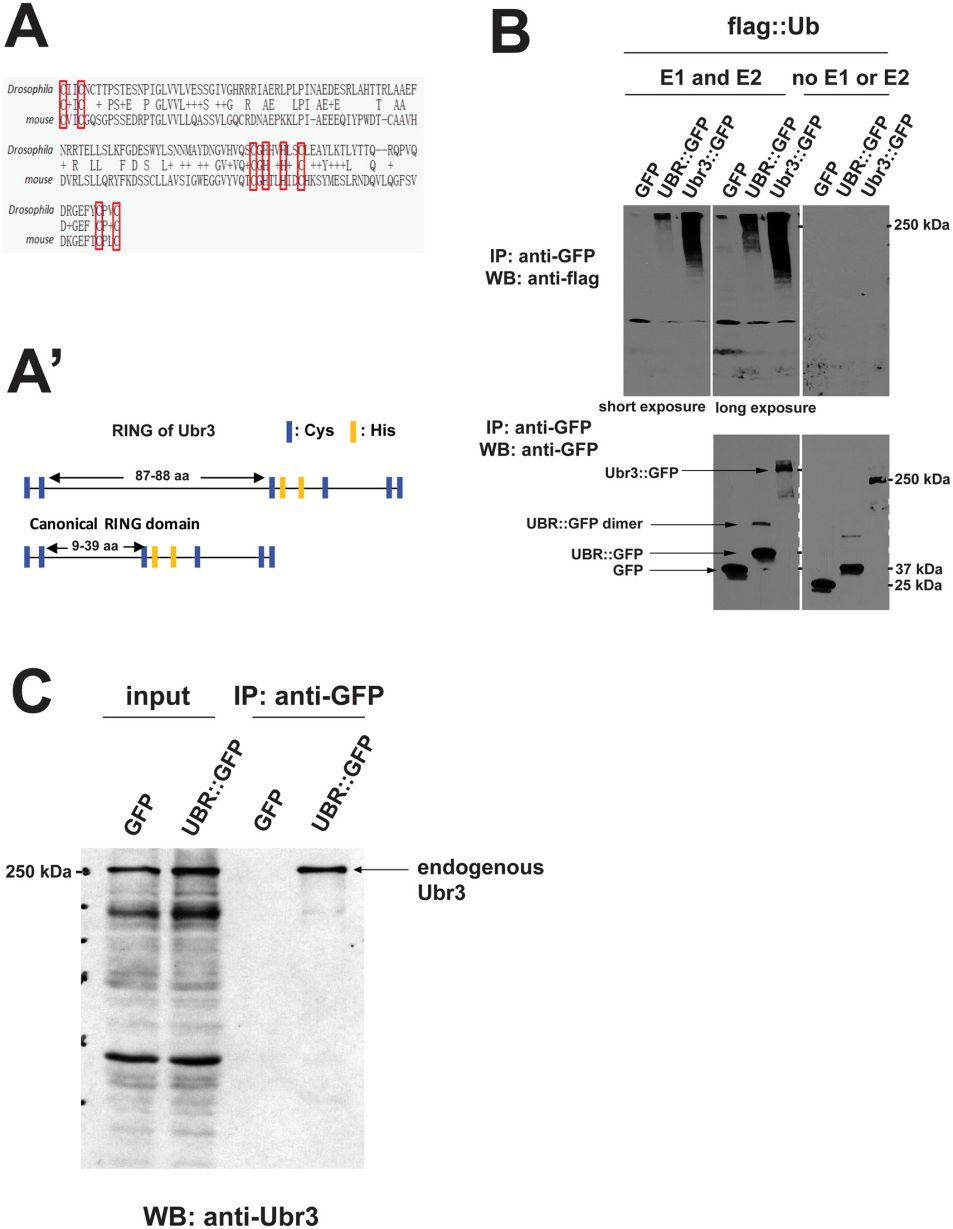
Ubr3 exhibits Ubiquitin E3 ligase activity and can ubiquitinate itself. (A-A’) Alignment of the RING domain sequences of Ubr3 in *Drosophila* and mouse. The Cys-X2-Cys-X87-88-Cys-X1-His-X2-His-X2-Cys-X23-25-Cys-X2-Cys motif (highlighted by red boxes) is somewhat divergent from the canonical RING domain sequence Cys-X2-Cys-X9-39-Cys-X1-3-Cys-X2-3-His-X2-Cys-X4-48-Cys-X2-Cys (X indicates any amino acid, Cys and His are exchangeable) [59]. (B-B’) Ubr3::GFP, UBR domain-GFP (UBR::GFP) and GFP control were immunoprecipitated from whole cell protein extracts of transiently transfected S2 cells under stringent conditions (see materials and methods) and subsequently incubated with E1/E2 conjugating enzymes and Flag::Ub to initiate self-ubiquitination reactions. Western blot analysis was performed to detect ubiquitinated proteins using anti-Flag antibodies. Ubr3 full length protein (lane 3) produces a smear with low motility, corresponding to ubiquinated proteins. The UBR domain alone gives a weak signal (lane 2), evident especially upon longer exposure of the film. The specificity of the reactions is evident by the absence of signal in GFP control samples (lane 1) or upon omitting E1 and E2 enzymes. The membranes were stripped and re-probed with anti-GFP antibodies to ensure the presence of the UBR::GFP or GFP. An additional band of ~80kDa is observed in UBR::GFP samples, which is approximately twice the size of UBR::GFP (arrow). (C) UBR domain interacts with endogenous Ubr3. UBR::GFP or GFP alone was immunoprecipitated from lysates of transiently transfected S2 cells using anti-GFP beads. Western blot was then performed with anti-Ubr3 antibody. Over-expressed UBR::GFP (lane 4), but not GFP alone (lane 3), co-immunoprecipitates with endogenous Ubr3 full length protein.

The RING domain of Ubr3 is not a canonical RING domain (Fig 4A and 4A’) [59]. To assess whether Ubr3 has E3 ligase activity, we performed an *in vitro* ubiquitination assay. Immunoprecipitation-purified Ubr3::GFP fusion proteins were incubated with E1 and E2 enzymes (HR6A) [39] and Flag-tagged Ubiquitin (Flag::Ub) peptides. Interestingly, Ubr3 poly-ubiquitinated itself, as shown in Fig 4B. Moreover, the UBR domain fragment may form a dimer when over-expressed, because a band of twice the molecular weight of GFP::UBR (~80 kDa) is detected (Fig 4B). Co-immunoprecipitation assays with the over-expressed UBR domain indicated that it interacts with the Ubr3 full-length protein present in whole cell extracts of S2 cells (Fig 4C). This suggests that Ubr3 interacts with the UBR domain of another Ubr3 molecule and that Ubr3 proteins poly-ubiquitinate each other.

### Ubr3 binds to and poly-ubiquitinates Cos2

**Fig 5:**
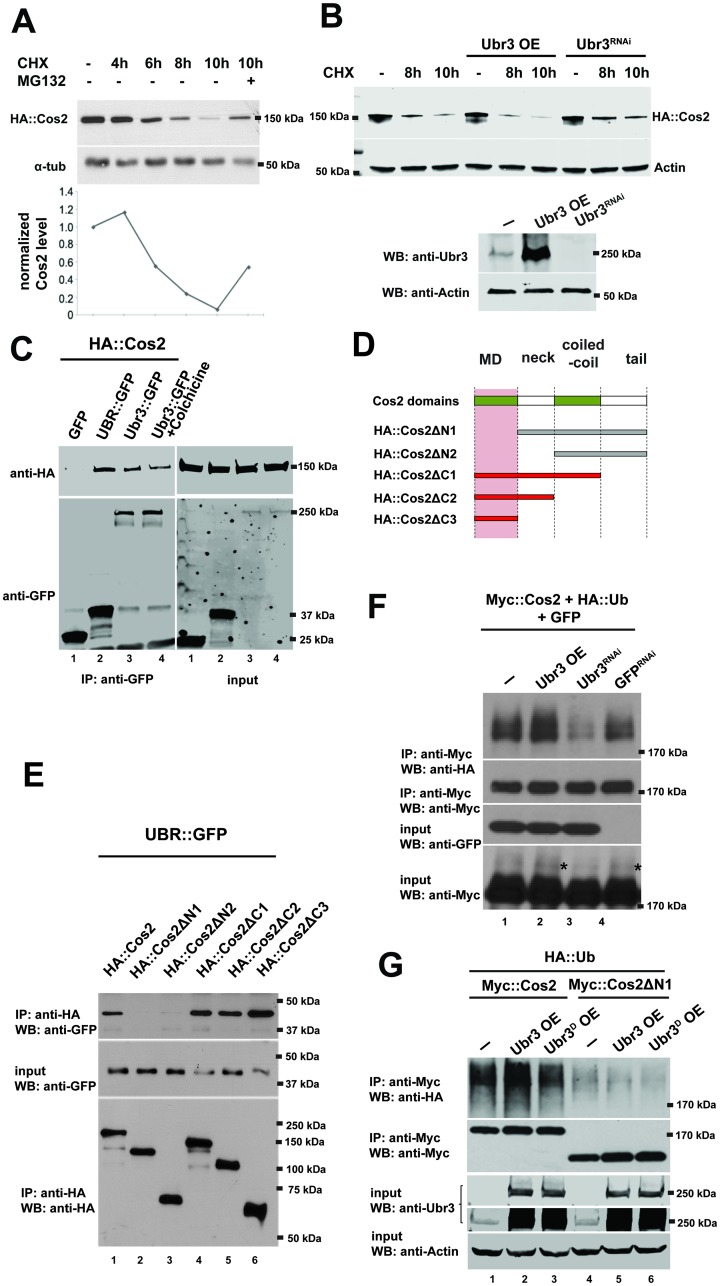
Ubr3 regulates the ubiquitination of Cos2. (A) S2 cells expressing HA::Cos2 fusion proteins were treated with CHX and MG132, or DMSO as a negative control, for the indicated time. The cell lysate was then examined by Western blot with antibodies. The HA::Cos2 signal intensity was normalized with α-tub and quantified in the lower panel. (B) S2 cells were transfected with Hh ligand and HA::Cos2 fusion proteins, and some cells were also co-transfected with Ubr3^RNAi^ or a construct to overexpress Ubr3. These cells were treated with Cyclohexamide for the indicated time. The cell lysate was then examined by Western blot (top panel). The levels of the Ubr3 protein were assessed with the anti-Ubr3 antibody. Anti-Actin served as loading control (bottom panel). (C) S2 cells were transfected with HA::Cos2 with GFP (lane 1) or UBR::GFP (lane 2) or Ubr3::GFP (lane 3) constructs. We performed co-immunoprecipitation assays with anti-GFP agarose followed by western blot assays on the cell lysates. Both UBR domain fragments (lane 2) and the Ubr3 full length protein (lane 3) co-precipitate with Cos2, whereas GFP (lane 1) does not. S2 cells co-transfected with HA::Cos2 and Ubr3::GFP were treated with Colchicine for 5 hours prior to harvest. Co-immunoprecipitation assay with anti-GFP agarose beads and western blots were performed from the cell lysate. (D) Schematic diagram of Cos2 deletion constructs. (E) Co-immunoprecipitation assays with lysates from S2 cells, transfected with UBR::GFP and the constructs indicated in C reveal that the UBR domain of Ubr3 interacts physically with full length Cos2 and Cos2ΔC1-ΔC3 (red bars in C, lanes 1, 4–6 in D), but not with Cos2ΔN1-ΔN2 (gray bars in B, lanes 2, 3 in D). (F) S2 cells were co-transfected with Myc::Cos2 and HA::Ub, in combination with Ubr3, Ubr3^RNAi^ or GFP^RNAi^. Following immunoprecipitation and Western blot analysis with anti-HA (upper panel), we found that Cos2 is ubiquitinated, as indicated by the smeary shift of the protein (lane 1). Over-expression of Ubr3 increases ubiquitinated Cos2 (lane 2), whereas down-regulation of Ubr3 through Ubr3 RNAi (lane 3) decreases ubiquitinated Cos2. GFP^RNAi^ was used as a negative control. The asterisks indicate ubiquitinated Cos2 which exhibits a higher molecular weight.(G) S2 cells were co-transfected with Myc::Cos2 or Myc::Cos2ΔN1, and HA::Ub, in combination with wild type Ubr3 or E3 dead form of Ubr3 (Ubr3^D^), following immunoprecipitation and Western blot analysis.

To test whether the up-regulation of Cos2 in *ubr3* mutant cells is due to defective degradation by the proteasome, we performed a degradation assay of Cos2 in *Drosophila* S2 cells. We found degradation of Cos2 proteins begins 6 hours after treatment with a translational inhibitor cycloheximide (CHX) and that the level of Cos2 decreased to 10% after 10 hours of treatment (Fig 5A). Addition of the proteasomal inhibitor MG132 suppressed the degradation of Cos2 (Fig 5A), suggesting that Cos2 proteins are degraded via the proteasome. The degradation of Cos2 is partially suppressed by down-regulation of Ubr3 by Ubr3^RNAi^ and promoted by over-expression of Ubr3 (Fig 5B), suggesting that Ubr3 mediates the degradation of Cos2.

Because ubiquitination is known to regulate protein abundance through proteasome-mediated degradation, Cos2 levels may be regulated via Ubr3-mediated ubiquitination. To determine whether Ubr3 interacts physically with Cos2 and to map which domains are required for this interaction, we performed co-immunoprecipitation assays. As shown in Fig 5C, both the UBR domain fragment and the full length Ubr3 protein interact with Cos2 (lane 2 and lane 3). To exclude the possibility that Cos2 binds to Ubr3 indirectly via microtubules, we treated S2 cells with the microtubule-destabilizing agent Colchicine. The Cos2-Ubr3 interaction is not affected by Colchicine treatment (lane 4 in Fig 5C), suggesting that Cos2 does not bind Ubr3 via microtubules. To identify which domain of Cos2 is critical for the interaction with Ubr3, we tested a series of deletion constructs of Cos2 (Fig 5D) in co-IP assays with the UBR domain. We found that only the fragments bearing the N-terminal motor domain (MD) of Cos2 (Cos2ΔC1, ΔC2, and ΔC3) interacted with the UBR domain (Fig 5E). Hence, Ubr3 binds to the N-terminal MD of Cos2 with its UBR domain.

To detect the ubiquitination of Cos2, we performed immunoprecipitation assays and examined the ubiquitination of Cos2 in S2 cells that express *ubr3* (Fig 4C). As shown in Fig 5F, the ubiquitinated Myc-tagged Cos2 (Myc::Cos2) was detected by an anti-hemagglutinin (HA) antibody in S2 cells co-transfected with an HA-tagged ubiquitin construct (HA::Ub; Fig 5F, lane 1, top panel). In addition, the HA signal exhibited a lower mobility shift compared to the major band detected by anti-Myc antibody (Fig 5F), indicating that these bands correspond to the ubiquitinated forms of Cos2. Over-expression of an E3 ligase dead form of Ubr3, in which the residues required for RING domain activity (Fig 4A) were mutated to alanines, did not cause an increase in ubiquitination of Cos2 (Fig 5G, lane 1–3), suggesting that the E3 ligase activity of Ubr3 mediates the ubiquitination of Cos2. In addition, removing the Ubr3 binding domain of Cos2, Cos2ΔN1, abolished most of the ubiquitination of full length Cos2 (Fig 5G, lane 4–6). The residual ubiquitination of Cos2ΔN1 may result from endogenous full length Cos2 that co-precipitates with Cos2ΔN1 through dimerization [10, 11].

To determine whether Ubr3 regulates Cos2 ubiquitination, we examined the levels of Cos2 ubiquitination when Ubr3 was either over-expressed or knocked down by RNAi. As shown in Fig 5F, the co-expression of Ubr3 with Cos2 increased Cos2 ubiquitination, whereas inactivation of Ubr3 by RNAi decreased ubiquitination (lane 2 and lane 3, top panel). A control GFP RNAi (negative control) did not significantly change the level of Cos2 ubiquitination (lane 4, top panel). These results suggest that Ubr3 interacts with and ubiquitinates Cos2.

### Hh signaling regulates Cos2 ubiquitination by Ubr3

**Fig 6:**
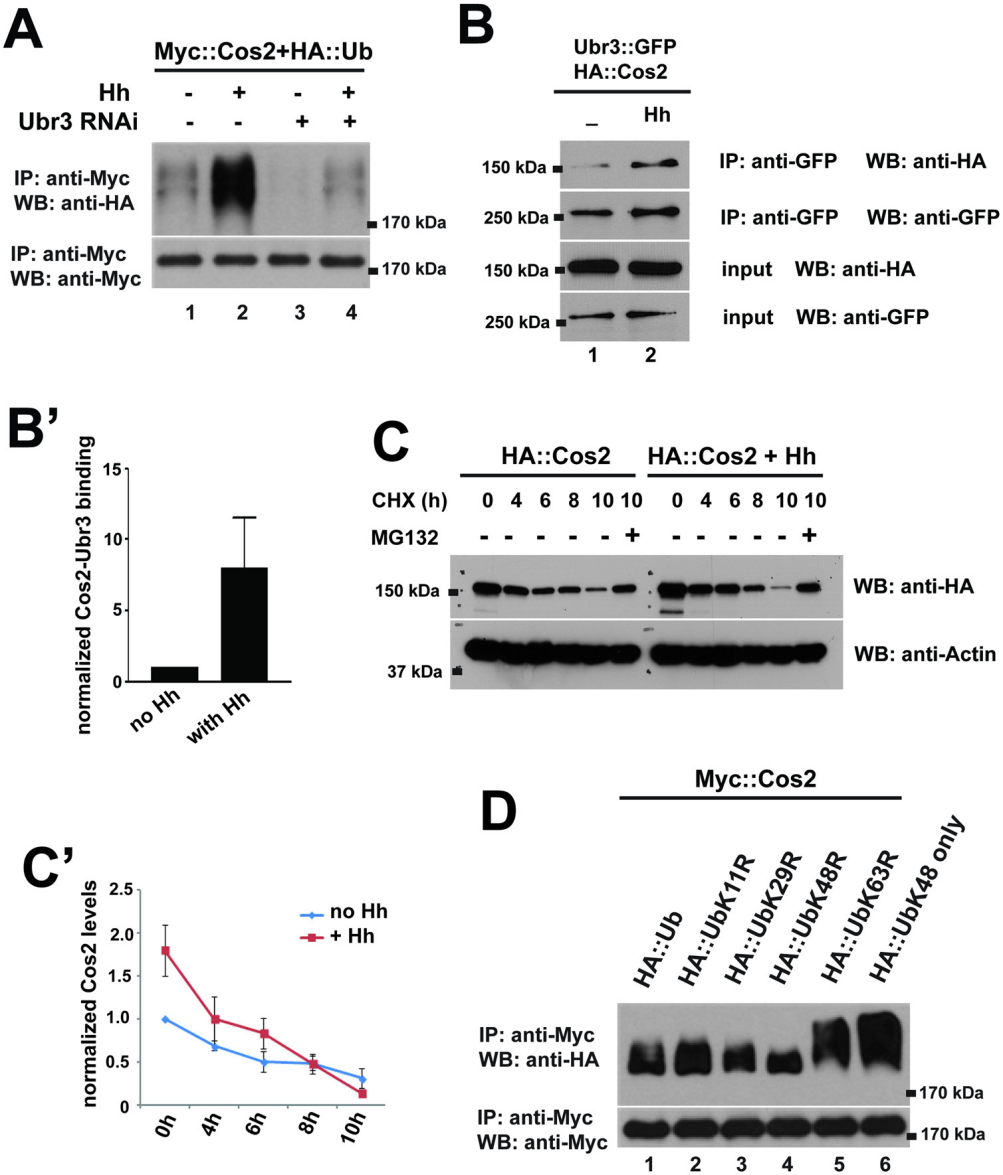
Ubr3-mediated poly-ubiquitination of Cos2 is tightly controlled by Hh activation. (A) S2 cells were co-transfected with Myc::Cos2 and HA::Ub, in combination with treatment of Hh-conditioned medium and with/without Ubr3 dsRNA. Immunoprecipitation assay was carried out with the indicated antibodies. Western blot with the anti-Myc antibody indicates the evenly-expressed Cos2. (B) S2 cells were co-transfected with Ubr3::GFP, HA::Cos2 and Hh (lane 2) or a control pUASTattB construct (lane 1). The cell lysate was immunoprecipitated with antibodies to the GFP tag and blotted with antibodies against GFP and HA. (C-C’) S2 cells expressing HA::Cos2 fusion proteins and Hh were treated with CHX, MG132, or DMSO as a negative control, for the indicated time. The cell lysate was then examined by Western blot with antibodies. Quantification of Cos2 levels were shown in C’. (D) S2 cells were co-transfected with Myc::Cos2 and HA::Ub or the indicated HA::Ub mutants. Immunoprecipitation was carried out with the anti-Myc antibody and Western blotted with the anti-HA antibody to detect the Cos2-bound HA::Ub.

We next tested whether Hh signaling regulates the ubiquitination of Cos2. Interestingly, we found that the ubiquitination of Cos2 was strongly enhanced by Hh treatment (Fig 6A, lane 2). This increased ubiquitination was abolished by down-regulation of Ubr3 (Fig 6A, lane 4), suggesting that Ubr3 mediates Hh induced ubiquitination of Cos2. This implied that Ubr3-mediated ubiquitination of Cos2 was tightly controlled by Hh signaling. Because Ci is not expressed in S2 cells, Hh-induced ubiquitination of Cos2 cannot be mediated by a positive, transcriptional feedback loop that depends on Ci. We therefore tested whether Hh may promote binding of Ubr3 to Cos2. We performed co-IP assays between the Ubr3 and Cos2 in the presence or absence of Hh. As shown in Fig 6B, the interactions between Ubr3 full-length protein and Cos2 (lanes 1 and 2 in Fig 6B) were strongly increased by Hh. These data show that Hh induces the ubiquitination of Cos2 by promoting the association of Ubr3 with Cos2. Consistent with Hh-induced poly-ubiquitination of Cos2, we also observed a faster degradation of Cos2 upon Hh treatment (Fig 6C and 6C’).

The ladder pattern of the HA signal in Fig 5E suggests that Cos2 is poly-ubiquitinated. We further determined the ubiquitination chain pattern by using a panel of ubiquitin mutant constructs [34]. Compared to wild-type ubiquitin (Fig 6D, lane 1, top panel), a mutated lysine 48 in ubiquitin (HA::UbK48R) abolished the formation of the ubiquitin chain (lane 4, top panel), whereas altered lysine 11 (K11R), lysine 29 (K29R), or lysine 63 (K63R) did not affect chain formation. In addition, mutating all of the lysine residues except lysine 48 (HA::UbK48 only) leads to longer ubiquitination chains (Fig 6D, lane 6, top panel). The single sharp band of Cos2 ubiquitination by K48R indicates a mono-ubiquitinated Cos2 that cannot be further elongated due to the lack of K48. Together, these data indicate that Cos2 undergoes K48-linked poly-ubiquitination.

### Ubr3 positively regulates Shh signaling in zebrafish

**Fig 7:**
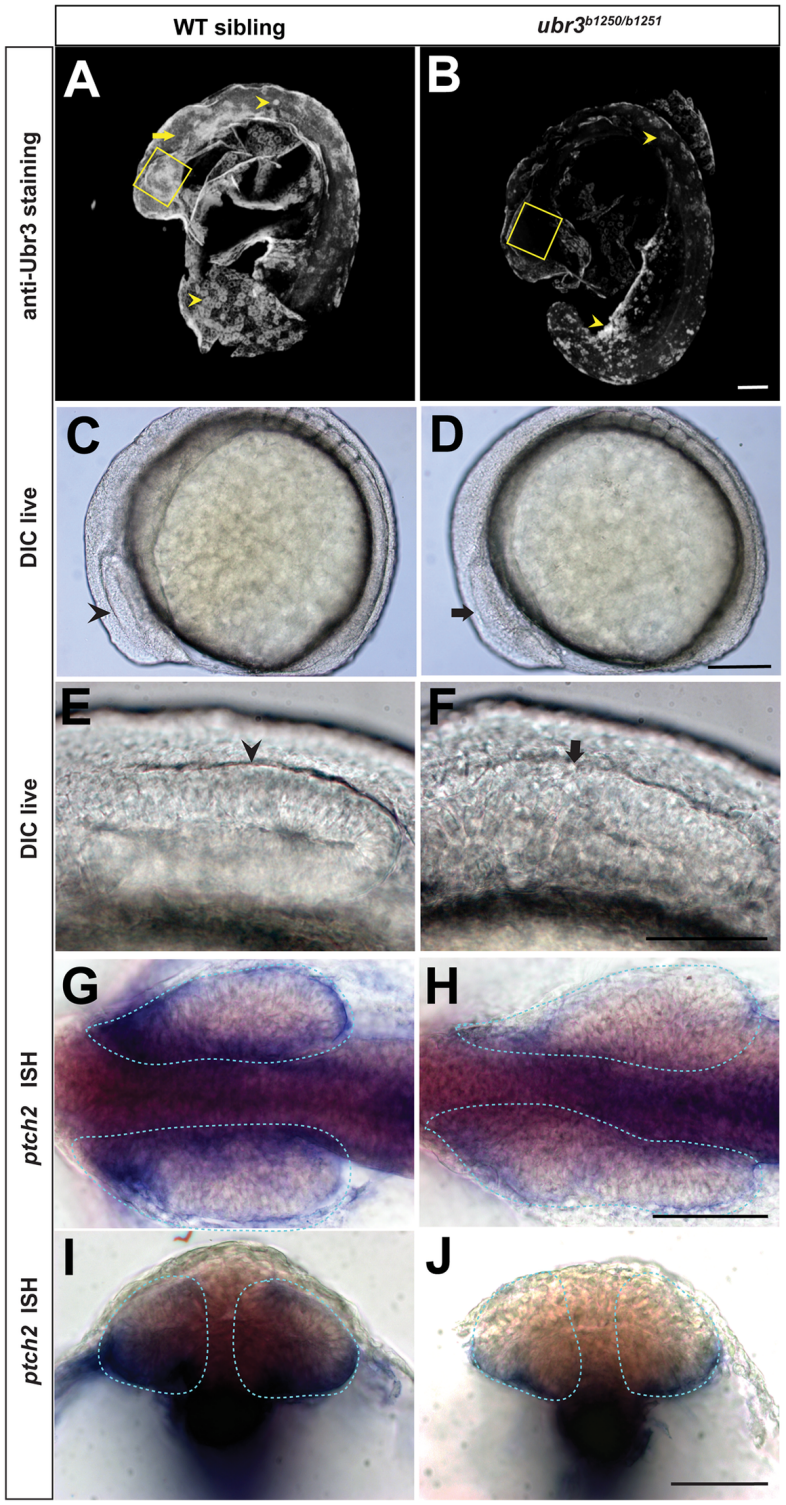
Ubr3 is required for Hh signaling and proper optic vesicle morphogenesis. (A-B) Lateral views of zebrafish embryos at 18-somites stage after removing the yolks, labeled with anti-Ubr3 antibody. Boxes outline the zebrafish retina, arrow points to central nervous system. The remaining signal in *ubr3*^*b1250/b1251*^ mutant corresponds to non-specific autofluorescence emanating from the yolk and some peridermal cells (arrowheads). (C-D) DIC (Differential Interference Contrast) imaging showing lateral views of 6-somites stage zebrafish embryos. (C) Wild-type optic vesicle (arrowheads) is morphologically visible and characterized by a stratified epithelial thickening. The cavitation seam divides the presumptive retina into dorsal and ventral halves. (D) *ubr3* trans-heterozygous mutant optic vesicle (arrow) has morphological defects characterized by disorganized tissue that lacks epithelial morphology. The cavitation seam is disrupted or absent. (G-J) In situ hybridization for *ptch2* in wild-type (G, I) and *ubr3* mutant (H, J) zebrafish embryos. Note the down-regulation of *ptch2* in *ubr3* mutant optic vesicles (dotted area). Dorsal views of flat-mounted zebrafish embryos. (G, H) Cross sections through the optic vesicle of embryos shown in G and H, respectively. All the scale bars represent 50μm.

To determine whether Ubr3 plays a conserved function in vertebrates, we created two independent zebrafish *ubr3* mutant alleles using CRISPR/Cas9. The *ubr3* gene is predicted to encode a protein of 1808 amino acids, and the *ubr3*^*b1250*^ allele lacks 28 nucleotides (Del 378–405) downstream of the predicted ATG (S4A Fig) leading to a frameshift and early stop codon. The mutant protein should encode only 129 amino acids (S4C Fig), lacking the UBR and RING domains. The second allele, *ubr3*^*b1251*^ carries a 4 nt insertion at position 220 (S4B Fig), also causing a frameshift and early stop codon. *ubr3*^*b1251*^ is predicted to encode a 78 aa protein lacking all functional domains (S4C Fig). Using an anti-Ubr3 antibody, we detected expression of Ubr3 in the developing retina, central nervous system and trunk, which are lost in *ubr3*^*b1250/b1251*^ mutant zebrafish (Fig 7A and 7B). Three independent crosses between single carriers heterozygous for the b1250 and b1251 alleles resulted in progeny with a distinguishable and reproducible retinal phenotype in a Mendelian frequency (f = 0.22, f = 0.27, f = 0.23, n = 270). At the 5-6-somites stage, phenotypically wild-type siblings display optic vesicles characterized by a compacted and stratified epithelium (Fig 7C, 7E and 7G). The optic vesicles of the *ubr3* trans-heterozygous mutants failed to form a cohesive and stratified epithelium (Fig 7D, 7F and 7H). Because appropriate levels of Sonic Hedgehog (Shh) signaling are essential for eye morphogenesis [60, 61], we examined the transcriptional levels of *ptch2*. In zebrafish, *ptch2* is a direct target of Shh signaling [62, 63]. In wild-type embryos, a gradient of *ptch2* expression was observed within the optic vesicle (dotted area in Fig 7G and 7I). This gradient was characterized by high levels of *ptch2*-expressing cells localized in the ventral border of the vesicle, and low level expressing cells localized in the dorsal border region and vesicle core (Fig 7I). In *ubr3* mutants, *ptch2* expression was strongly decreased (Fig 7H and 7J). Consistent with decreased Shh signaling, *ubr3*^*b1250/b1251*^ trans-heterozygous mutants show a 30% increase in the angle of the somite in comparison with phenotypically wild-type siblings (S5A, S5C and S5E Fig). The opening of the somite angle is a common morphological phenotype of mutants with reduced Hedgehog signaling [64–67]. In addition, we observed a gain of Rohon-Beard sensory neurons at the level of the posterior central nervous system (CNS) in *ubr3* mutants (S5B and S5D Fig), assessed by expression of a Rohon-Beard sensory neuron marker *islet2* [68]. Because Hedgehog restricts CNS dorsal fate acquisition [69], this result supports the interpretation that Hedgehog signaling is decreased in *ubr3* mutants. This finding is also consistent with our observation of decreased retinal *ptch2* expression in the absence of *ubr3* (Fig 7H and 7J). Because Kif7-depleted zebrafish embryos do not show de-repression of Hh target genes in the CNS [27], our findings further suggest that, at least in zebrafish, Ubr3 may regulate not only Kif7 but also other intracellular negative regulators of Hedgehog signaling in the CNS. Different zebrafish Hh signaling mutants show distinct degrees of severity, highlighting the tissue-specific requirements of Hh levels during development [60, 70–74]. Similarly, loss of *ubr3* does not result in cyclopia or inner ear defects, showing that these mutants have a less severe phenotype when compared to *smoothened* mutant animals. Hence, *ubr3* zebrafish mutants retain some residual Hh signaling. Thus, our data show that Ubr3 positively regulates Hedgehog signaling in tissues sensitive to high levels of Hh like the mesoderm and neuroectoderm. In addition, the transcription of *ubr3* is strongly reduced in *smo*^*hi1640-/-*^ mutant animals [70], which lose Shh activity (S5F and S5H Fig). In contrast, ectopic activation of the Shh pathway by injection of the mRNA encoding a dominant negative form of PKA (dnPKA) [75] expands the expression domain of Ubr3 (S5G and S5I Fig). These data suggest that Shh signaling promotes the transcription of *ubr3* in zebrafish, similar to what we observed in Drosophila. In summary, Ubr3 is required for the transduction of Hh signaling and proper eye morphogenesis in zebrafish.

### UBR3 negatively regulates protein level of Kif7 through poly-ubiquitination in mammalian cells

**Fig 8:**
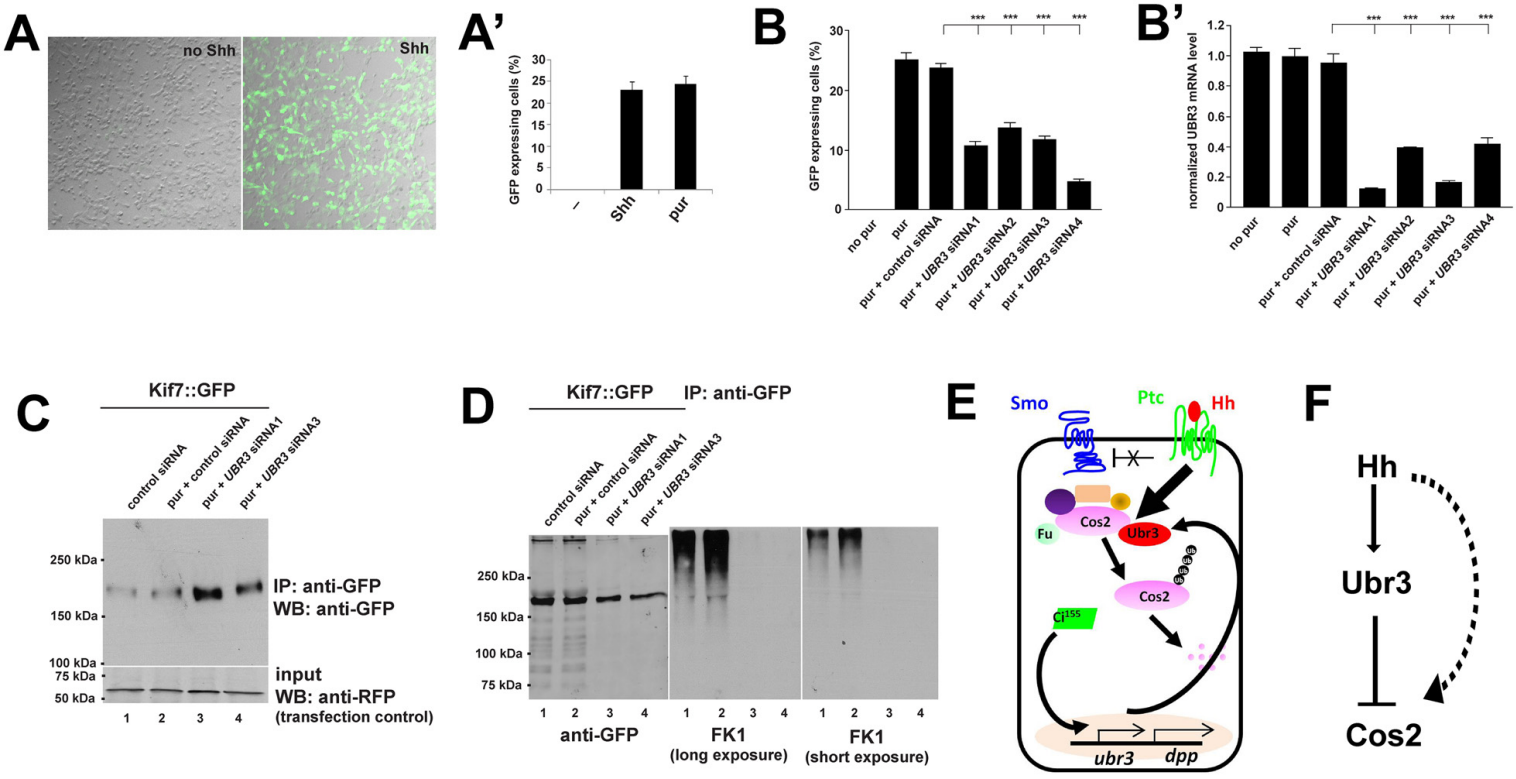
UBR3 affects Shh signaling in mammalian cells by poly-ubiquitinating Kif7. (A) The Gli::GFP reporter is induced by both Shh and purmorphamine (pur) in C3H10T1/2 cells. (A’) Quantification of Gli::GFP expressing cells in response to Shh and pur. (B-B’). (B) Quantification of the proportion of Gli::GFP expressing C3H10T1/2 cells. (B’) Real time PCR to estimate the expression of UBR3 under the conditions shown in B. All values have been normalized to cells treated with pur and transfected with no siRNA. (C) C3H10T1/2 cells were transfected with the Kif7::GFP expression construct. Cells were transfected with various siRNAs and treated with pur (lanes 2–4) or DMSO (lane 1) and lysed, followed by immunoprecipitation against GFP. Western blots were performed with indicated antibodies. RFP expressions provided as transfection controls. (D) Kif7::GFP was immunoprecipitated from the lysate of the cells transfected with Kif7::GFP construct and shown siRNA followed by treatment of MG132. FK1 anti-ubiquitin antibody was used to detect ubiquitinated Kif7 in a Western blot. (E) A model shows the regulation of Hh signaling by Ubr3. Ubr3 regulates the proteasomal degradation of Cos2 via K48-mediated poly-ubiquitination of Cos2. *ubr3* is one of the target genes activated by Hh signaling, forming a positive feedback to promote further activation of Hh signaling. Hh signaling also promotes physical association between Ubr3 and Cos2, increasing the ubiquitination of Cos2. (F) Diagram shows parallel regulations of Cos2 protein levels downstream of Hh activation.

To test whether UBR3 also plays a role in Shh signaling in mammals, we used C3H10T1/2 mouse mesenchymal cells. These cells respond to Shh and activate Shh target genes [76]. We first confirmed that Ubr3 is expressed in C3H10T1/2 cells by RT-PCR (see Fig 8B’). We then infected these cells with a lentivirus bearing 7 tandem binding sites for Gli (the vertebrate homologue of Ci) that control the expression of a GFP reporter. Addition of either the Shh ligand or purmorphamine, an agonist of Smo [77], to C3H10T1/2 cells induced GFP expression in about 25% of the cells (Fig 8A and 8A’). To determine whether knockdown of UBR3 impairs Shh signaling, we measured the proportion of GFP-expressing C3H10T1/2 cells transfected with one of four different siRNAs against UBR3 or a scrambled siRNA control, followed by purmorphamine treatment. Induction of the Gli::GFP reporter by purmorphamine was suppressed when siRNA reduced the UBR3 levels (Fig 8B), as judged by real time PCR (Fig 8B’). In addition, down-regulation of UBR3 resulted in up-regulation of Kif7 (Fig 8C), the mammalian homolog of Cos2. To assess poly-ubiquitination of Kif7, we purified Kif7 through immunoprecipitation and loaded the Western blot lanes with equal amounts of protein (unlike in Fig 8C where we loaded equal amounts of cells). We observed decreased poly-ubiquitination of Kif7 upon knockdown of UBR3 (Fig 8D). These data indicate that UBR3 regulates Shh signaling through poly-ubiquitination of Kif7 in vertebrate cells, a process that seems to be evolutionarily conserved.

## Discussion

Numerous studies have shown that Cos2 plays a central role in Hh signaling [10, 11, 22, 25, 26, 78, 79]. Cos2 is both necessary and sufficient to regulate Ci [11, 22] and the level of Cos2 protein is critical for activating Hh signaling [80, 81]. Here, we identified Ubr3 as a novel regulator of Cos2 in a forward genetic screen in *Drosophila* and showed that this gene is conserved in vertebrates and affects Hh signaling. We present evidence that the level of Cos2 protein is tightly controlled through a Ubr3-mediated poly-ubiquitination pathway (Fig 8E).

Although most of the core components of Hh signaling are evolutionarily conserved, there are differences in Hh signaling between vertebrates and invertebrates [82]. For example, Cos2 can be phosphorylated by the kinase Fused [30, 31], but the kinase that phosphorylates Kif7 remains to be identified, because mice lacking Fused have no apparent defects in Hh signaling [83, 84]. Given that a Kif7 phosphatase affects Hh signaling in vertebrates [85] it is likely that phosphorylation of Kif7 is important even if these sites are different than those observed in Cos2. Here, we present the first evidence that the levels of Cos2 and Kif7 proteins are also controlled by poly-ubiquitination via a conserved Ubr3 E3 ligase. The conservation of this mechanism is supported by the finding that fly Cos2 rescues the Kif7 mutant phenotypes in zebrafish [27]. Although we have shown that the degradation of Cos2 protein is regulated by Ubr3 mediated ubiquitination, the increased Cos2 proteins in *ubr3* mutant cells may also result from up-regulated transcription of Cos2.

Although Hh promotes poly-ubiquitination and degradation of Cos2 (Fig 6A and 6C), we did not observe a decrease of Cos2 proteins at the morphogenetic furrow, where Hh signaling is activated. Instead, the level of the Cos2 protein is modestly elevated when compared to surrounding tissues/cells (S6A and S6A’ Fig), consistent with a previous finding [11]. We also observe that activation of Hh in S2 cells up-regulates Cos2 (Fig 6C). The observation that activation of Hh signaling promotes the degradation of Cos2 and that Cos2 protein level is increased, but not decreased by Hh activation, suggests that some mechanism other than ubiquitination up-regulates the level of Cos2 protein (Fig 8F). Ubr3-mediated degradation of Cos2 may function as a mechanism to prevent aberrantly high levels of Cos2, thereby toning down Hh signaling. This may also underlie the observation that not all cells that require Hh signaling are affected in flies. This hypothesis is also supported by the finding that loss of *ubr3* in zebrafish affects developmental processes that rely on high levels of Shh signaling but does not affect those that respond to low Shh signaling (Fig 7 and S4 Fig).

Cul1 functions downstream of Cos2 to process Ci^155^, one would anticipate that Cul1 is epistatic to Cos2. This is inconsistent with the observation that down-regulation of Cul1 in *ubr3* clones in the morphogenetic furrow of *Drosophila* eye discs fails to restore Ci^155^ expression (Fig 3E), whereas down-regulation of Cos2 restores Ci^155^ levels (Fig 3D). This may be because the RNAi expression does not deplete the protein sufficiently, or because, Cos2 may regulate Ci^155^ through a mechanism independent of Cul1.

Although our data clearly show that Ubr3 plays a role in Hh signaling at the morphogenetic furrow, we do not observe a loss of Ci^155^ in *ubr3* mutant clones in wing discs (S6B and S6B’ Fig). However, we observed a similar up-regulation of Cos2 in *ubr3* mutant clones in wing discs (S6C and S6C’ Fig), implying that Ubr3 mediated poly-ubiquitination of Cos2 may be present in wing discs. The lack of a Hh phenotype in posterior compartment cells of wing discs may be due to another E3 ligase that is functionally redundant and downregulates Cos2. Alternatively residual Ubr3 in *ubr3* mutant cells due to perdurance of Ubr3 products may partially downregulate Cos2, allowing activation of Hh signaling. When we sensitized the background by over-expressing *ptc*^*DN*^ to ectopically activate Hh signaling, we find that loss of *ubr3* strongly suppresses the activation of Hh signaling in clones, gauged by the reduced clone sizes and Ci^155^ levels (S6D–S6E’ Fig). This may also be the reason why not all tissues display the typical Shh phenotype in zebrafish. In addition, ectopic activation of Hh signaling leads to up-regulated transcription of *ubr3* (S6F–S6J Fig), suggesting that the positive feedback of Ubr3 is present in the wing.

Hh signaling shares many similarities with Wnt signaling [86]. Both pathways regulate many developmental processes and induce human cancers when the pathways are aberrantly activated. Moreover, the principal signaling mechanisms are based on similar features. Each pathway is activated through ligand binding of a G-protein coupled receptor, leading to the downstream activation of a transcription factor through phosphorylation-dependent proteolysis. Axin is the scaffold protein that recruits an activation complex in Wnt signaling, which mediates phosphorylation of β-catenin [87]. This function is similar to that of Cos2 in Hh signaling. Interestingly, previous studies have shown that the levels of Axin protein are also regulated by an E3 ligase, RNF146, through poly-ubiquitination [88–90]. Upon activation of Wnt signaling, Axin undergoes tankyrase-dependent poly ADP-ribosylation, which promotes RNF146-Axin interaction [89]. Ubr3 seems to regulate the poly-ubiquitination of Cos2 in a similar manner, given that Hh activation promotes the Ubr3-Cos2 interaction and the ubiquitination of Cos2. Hence, our data suggest further similarities between the Hh and Wnt signaling pathways.

## Materials and Methods

### Fly strains and genetics

*ubr3*^*A*^ and *ubr3*^*B*^ mutants were isolated in a forward genetic screen as previously described [45, 48]. *y w ubr3*^*A*^ *FRT19A/FM7c Kr-Gal4*, *UAS-GFP* and *y w ubr3*^*B*^ *FRT19A/FM7c Kr-Gal4*, *UAS-GFP* flies were crossed to, *y w tub-Gal80*, *eyFLP*, *FRT19A; actin-Gal4*, *UAS-CD8*::*GFP/CyO* and *y w UbxFLP*, *tub-Gal80 FRT19A*; *UAS-CD8*::*GFP*, *actin-Gal4* to generate GFP-labeled *ubr3* homozygous mutant clones using the MARCM technique [91]. The *ubr3* genomic rescue transgenic fly strain was generated using the P[acman] system, BAC recombineering and transgenic platform developed in our laboratory [92]. *ubr3* cDNA transgenic flies were generated through φC31-mediated transgenesis [92]. Additional strains used in the study are as follows: *dpp-lacZ* [93], *Df(1)BSC622* [[94], Bloomington *Drosophila* Stock Center], *P[lacW]CG42593*^*G0307a*^ [[54], Bloomington *Drosophila* Stock Center] *cul1*^*EX*^, *FRT42D/CyO* [58], *FRT42D/CyO* [95], *UAS-p35* (a kind gift from Andreas Bergmann), *eyg-Gal4* [96], *UAS-cos2/CyO* [81], *UAS-cul1/CyO* [58], *UAS-ubr3*^*RNAi*^ [P{GD12698}, [97], Vienna *Drosophila* Resource Center] *UAS-cos2*^*RNAi*^ [[97]; Vienna *Drosophila* Resource center], *UAS-cul1*^*RNAi*^ [TRiP. HM05197, [98]; Bloomington *Drosophila* Stock Center]; *UAS-Ci*^*RNAi*^ [TRiP.JF01272, [98]; Bloomington *Drosophila* Stock Center]*UAS-ptc*^*DN*^ (a kind gift from Michael Galko). All flies were maintained on standard food at 25°C.

### Zebrafish strains and husbandry

Zebrafish strains were AB wild-type, *ubr3*^*b1250*^, *ubr3*^*b1251*^ and *smo*^*hi1640*^. The *ubr3* mutations are recessive alleles. Phenotypically wild-type siblings were used as controls and labeled as wild type in Fig 7. Animals were raised in a 10 hour dark and 14 hour light cycle and maintained as previously described [99]. Embryos were staged according to the standard series [100]. All animal use protocols were IACUC-approved.

### CRISPR mutagenesis and genotyping

CRISPR mutagenesis was carried as previously described with minor modifications [101, 102]. The zebrafish *ubr3* reference sequence used in this study was XM_009304449.1. Identification of target sequences was done using Zifit software [103, 104]. Candidate sequences were then blasted against the zebrafish genome (Zv9) and those with unique hits were selected. The following target sequences were selected b1250: 5’- GGGGCCTGTGACTGCGGGGA-3’, located in the sense strand, and b1251: 5’-GGCGTTATCGTAGGATCGGA3’, located in the antisense strand (Fig 7, S1 Fig). A guide RNA (gRNA) template was created by PCR. A T7 promoter site was incorporated in the gene specific oligonucleotide, followed by the target sequence and the start of the guide RNA sequence (5’-gttttagagctagaaatagc-3’). The complementary guide RNA scaffold oligonucleotide sequence used was 5’-gatccgcaccgactcggtgccactttttcaagttgataacggactagccttattttaacttgctatttctagctctaaaac-3’. PCR was performed using Phusion polymerase (NEB) following the manufacturer’s recommendations. 10μM of each primer was used for the reaction. The first denaturation step was carried out at 98°C for 30 sec, followed by 40 cycles of denaturation at 98°C for 10 seconds, annealing at 60°C for 10 seconds, and extension at 72°C for 15 seconds. A final extension step was introduced at 72°C for 10 minutes. PCR products were purified using a PCR purification kit (QIAGEN). RNA was transcribed using a MEGAscript T7 kit following the manufacturer recommendations. A volume of 2nl of Cas9 RNA and gRNA were co-injected at a concentration of 100ng/μl each.

Screening of F0 founders and genotyping of F1 carriers were done by PCR and sequencing using the following primers: Primer b1250F (position 101–119): 5’-CTGCAGGAACTGCTGGATAG-3’; Primer b1250R (position 415–433): 5’-ACCCGCTCTCTCTCATCAC-3’. Primer b1251F (position75-94): 5’-TGACAACAGTTCAGGCTTGC-3’; Primer b1251R (position326-345): 5’-GTGGCGTTATCGTAGGATCG-3’.

### Embryo manipulation and RNA injection

250 pg of RNA encoding for a dominant negative regulatory subunit of the Protein Kinase A (dnPKA) [75] was injected into 1 cell stage embryos. dnPKA construct was linearized with NotI and transcribed with SP6 using a mMessage mMachine kit following the manufacturer´s recommendations. Embryos were fixed at 27hpf and processed for in situ hybridization against *ubr3*.

### Immunolabeling and imaging

Fly tissues were dissected in phosphate-buffered saline (PBS) at room temperature and fixed with 3.7% formaldehyde in PBS for 20 minutes, followed by permeabilization with 0.2% Triton-X100 in PBS. The primary antibodies and secondary fluorescently-labeled antibodies used were: chicken anti-GFP (1:1000, Abcam), rat-Elav [1:1000, 7E8A10, DSHB, [51], guinea pig anti-Sens [1:1000, [50]], rat anti-Ci [1:50, 2A1, DSHB [105]], rabbit anti- β-galactosidase (lacZ; 1:1000, Abcam), guinea pig anti-Ubr3 (1:1000, this study, see below), mouse anti-Cos2 [1:50, 17E11, DSHB [18]], mouse anti-Ptc [1:100, DSHB [106]], mouse anti-Fu [1:100, DSHB [18]], rabbit anti-Cul1 [1:250, [107]], rabbit anti-GM130 (1:500, Abcam), rabbit anti-Rab5 (1:500, Abcam), rabbit anti-Rab7 (1:500) [108], mouse anti-Rab11 (1:100, BD Biosciences) [109], mouse anti-Complex V (1:500) [110], ER-GFP (1:1000 incubate with cells over night, CellLight ER-GFP, BacMam 2.0. Thermo Fisher Scientific), PNA-biotin (Vector Laboratories). Alexa488-, Cy3- and Cy5- or DyLight649 conjugated affinity purified donkey secondary antibodies (1: 500, Jackson ImmunoResearch Laboratories) and DAPI (0.5 μg/ml, Life Technologies).

Zebrafish immunolabeling was performed as previously described [111] with the following minor modifications. 18-somites stage embryos were fixed in BT-fix overnight at room temperature. Embryos were permeabilized in PBS+1% Tween20 for 5 hours at room temperature. Anti-UBR3 antibody (Sigma Prestige, catalogue #HPA035390) was diluted in 1/500. Biotinylated anti-rabbit was used at 1/500. To detect signal, ABC kit (Vectorlabs) was used. A and B reagents were mixed together at an 1/100 dilution in PBS- Block and pre-incubated for 20 minutes at room temperature, then added to the samples for 25 minutes. Tyramide from the TSA kit (Perkin-Elmer) was diluted 1:50 in pre-warmed buffer reagent, and added to samples for 20 minutes following the manufacturer’s recommendations. The detection reaction was stopped by adding cold PBS+0.1%Tween20, followed by 4 washes in PBS+0.1%Tween20.

### Microscope image acquisition

Images were acquired using LSM510 and LSM710 confocal microscopes (Zeiss) and examined and processed using LSM viewer (Zeiss), ZEN (Zeiss) and Photoshop (Adobe) software. Immunostained zebrafish embryos were immersed in Vectashield, mounted laterally in a slide chamber and imaged with a Zeiss LSM5 confocal microscope. Live embryos were mounted laterally in 3% methylcellulsose and imaged on a compound microscope using DIC. ISH treated embryos were dissected in 90% glycerol, flat-mounted in 100% glycerol in a slide chamber and imaged on a compound microscope using DIC.

### Cloning, plasmid constructs and antibody production

A *ubr3* genomic rescue construct was constructed by cloning a 18.3 kb fragment of genomic DNA that contains the *ubr3* gene (X: 7,935,666… 7,953,967) [Release 6 *Drosophila* reference genome, [112]] into P[acman] [92, 113]. *ubr3* cDNA was constructed from exon sequences and cloned into pUASTattB using a GENEART Seamless Cloning and Assembly Kit (Life Technologies). GFP was tagged to the carboxyl terminus of the full length *ubr3* sequence or a partial sequence encoding only the UBR domain (aa 222–292). The flag sequence was conjugated to the carboxyl terminus of the full length *ubr3* sequence in the primer. Ubr3::flag was then amplified through PCR and cloned into pUASTattB through XhoI and XbaI. To generate E3 dead form of flag tagged Ubr3 expression construct, mutations results in all residues shown in red box in Fig 4A changed to alanines were introduced through synthesized DNA which spans 500 bp downstream from RsrII. This synthesized DNA fragment was then cloned together with PCR amplified flag tagged carboxyl fragment of Ubr3 into pUASTattB-Ubr3-flag through RsrII, BbsI and XbaI. The HA::Cos2N1 to 3 constructs were cloned into pUASTattB through EcoRI and XbaI. HA::Cos2ΔN1-2, HA::Cos2ΔC1-3 have been described [114]. Myc::Cos2 was constructed by fusion of 5xMyc tags to the N-terminus of the Cos2 coding sequence. The HA::Ub transgene has been described previously [34]. Kif7::GFP construct is a gift from Dr. Chi-Chung Hui [78].

The 7Gli:GFP reporter of Hedgehog signaling activity contains 7 repeats of the Gli binding site (5’-TCGACAAGCAGGGAACACCCAAGTAGAAGCTC) followed by GFP [115]. The primer pair (forward 5’-TGAAGCTTGCATGCCCTGCAGGACAAGCAGGGAACGCCCAAGTAG and reverse 5’ CTCGAGTACCGGATCCATTATATACCCTCTGCAGACTTGGGTGTTCCCTGCTTGTCG) was used to amplify the Gli binding sequences from the 8Gli-Luc plasmid by PCR. The reverse primer also contains a TATA sequence, which was used to rebuild the TATA box after the Gli binding sites. The destination plasmid pRRL.sin-18.ppt.TCF/LEF:GFP.pre [116] was linearized by PstI and BamHI digestion to cut out the TCF/LEF sequence and the TATA box. The resulting products were recombined into a linearized destination plasmid by infusion cloning according to the manufacturer’s protocol (Clontech).

For Ubr3 antibody production, the sequence encoding aa 751–1500 of Ubr3 was cloned into pET21 expression construct and expressed in *E*. *coli*. Purified inclusion bodies were used to immunize guinea pigs.

### In situ hybridization

*ubr3* in situ hybridization probes I and II anti-sense sequences contain 2558 to 3576 nt and 3775 to 4770 nt of *ubr3* cDNA, respectively. Anti-sense sequences were cloned into pGEM-T vector (Promega). Before transcription, the construct was linearized by SalI. *ubr3* RNA in situ probes were transcribed and labeled with a digoxigenin [113] RNA labeling kit (Roche). In situ hybridization to whole-mount discs was performed as previously described [117]. Probe I was used in images shown in Fig 2F, 2G, 2H and 2I and S6J Fig. Probe II was used in images shown in Fig 2E and 2J and S2I, S6F, S6H and S6I Figs.

Zebrafish whole-mount in situ hybridization was carried out as described with minor modifications [118]. Digoxigenin-labeled probes were prepared according to manufacturer´s instructions (Roche). Probe signal was detected using NBT/BCIP mix (Roche). The *ptch2* probe was kindly shared by Stone Elworthy (University of Sheffield, UK).

### Cell culture assays

S2 cells were cultured at 25°C in Schneider’s medium (Life Technologies) plus 10% heat-inactivated fetal bovine serum (Sigma), 100 U/mL penicillin (Life Technologies), and 100 μg/mL streptomycin (Life Technologies). Cells were split every 3 days and plated at a density of 10^6^ cells/well in 12-well cell culture plates for experiments. Transfections were carried out using Effectene transfection reagent (Qiagen). Ubr3 dsRNA was synthesized against nt 652–1,191. dsRNAs transfection and dsRNA against GFP have been described [114]. CHX (100 μM, Sigma) and MG132 (50 μM, Sigma) in dimethyl sulfoxide (DMSO) were added to S2 cells 48 hours after transfection and incubated for indicated time. An equal amount of DMSO was added as a negative control (-). 1ug/ml Colchicine (sigma) was incubated with cells for 5 hours before harvest.

C3H10T1/2 cells were cultured at 37°C in 5% CO_2_ in air in Eagle's Basal medium with Earle's BSS, 2 mM L-glutamine, 1.5 g/L sodium bicarbonate and 10% fetal bovine serum, as described by the ATCC (http://www.atcc.org/). Cells were split when reaching 80–90% confluence. Cells were transduced with lentivirus containing Gli::GFP reporter construct for 16h and then plated on 12-well cell culture plates. On the second day, siRNAs were incubated with Lipofectamine RNAiMAX reagent (Invitrogen) overnight. The sequences of siRNAs against *ubr3* are as follows: siRNA 1: GTTATAGCTTTGAATCAGT; siRNA 2: CAGAGTTTGCCTCACGACA; siRNA 3: CAAGATTGGTTTGATGCTA; siRNA 4: CAGAAATTGCTCGCAGAGT. Stealth RNAi^™^ siRNA Negative Control Med GC Duplex (Invitrogen) was used as control siRNA. 3μg/ml Shh (R&D systems) or 10 μM purmorphamine (Calbiochem) in culture medium, or the same amount of vehicle in culture medium for uninduced controls, was added to cells on the third day. Cells were photographed for GFP fluorescence and harvested 48 h after purmorphamine induction. The number of GFP-positive cells was manually counted and statistical testing was performed with a one way ANOVA followed by Dunnett’s test using uninduced cells as a control.

### Quantitative real-time PCR

For RNA extraction, total RNA from C3H10T1/2 cells was isolated by using Absolutely RNA miniprep Kit (Agilent Technologies). cDNA was synthesized using Superscript III First Strand Synthesis System for RT-PCR (Invitrogen). Quantitative real-time PCR (qPCR) was conducted with a Master SYBR Green kit (Applied Biosystems) and gene-specific primer sets on a Step One Plus real-time PCR system (Applied Biosystems). Each experiment was performed with three biological sample repeats and each PCR was performed in triplicate. L19 was used as an endogenous reference. The gene-specific primer sets used were as follows: L19 (RpL19): 5’-GGTCTGGTTGGATCCCAATG-3’ and 5’-CCCGGGAATGGACAGTCA-3’; UBR3: 5’-CTGATTCATAGAGGAGGCAG-3’ and 5’-ATGGAACAGCTGATTCAGAC-3’.

### Co-immunoprecipitation and western blot

S2 cells were lysed 48 h after transfection with plasmids in lysis buffer (Tris-HCl 25mM, pH 7.5, NaCl 150 mM, EDTA 1mM, NP-40 1%, Glycerol 5%, DTT 1mM) plus Complete proteinase inhibitor (Roche) for 30 minutes on ice, followed by centrifugation. In these experiments to detect the ubiquitination of Cos2 (Figs 5F, 5G and 6A), we treated S2 cells with 50 uM of MG132 24 h before harvesting the cells. The supernatant was then immunoprecipitated with agarose beads conjugated to antibodies recognizing different epitope tags, which had been previously equilibrated with lysis buffer, overnight at 4°C. The beads were then washed 3 times in washing buffer (Tris-HCl 10mM, pH 7.5, NaCl 150mM, EDTA 0.5 mM) before boiling in loading buffer. Western blotting was then performed with each sample. The following beads were used for immunoprecipitation: Chromotek-GFP-Trap Agarose Beads (Allele Biotechnology), Monoclonal Anti-HA−Agarose antibody (Sigma). Protein A resin and anti-Myc (9E10, Santa Cruz) were used for Myc immunoprecipitation. To examine the levels of Cos2 ubiquitination, a denaturing method was used as previously described [34]. Briefly, S2 cells were transfected with Myc::Cos2 and then lysed with denaturing buffer (1% SDS, 50mM Tris, pH 7.5, 0.5 mM EDTA, and 1 mM DTT) and incubated at 100°C for 5 min. The lysates were then diluted 10-fold with regular lysis buffer containing 1.5 mM MgCl_2_ and subjected to immunoprecipitation with the anti-Myc antibody. The proteins were then resolved on an 8% SDS-PAGE, and an immunoblot was performed using an anti-HA antibody to detect the HA::Ub or HA::Ub mutants. The antibodies used in Western blot analysis are as follows: anti-GFP (1:1000 Zymed or 1:1000, Millipore), anti-Myc (1:5000, 9E10, Santa Cruz), anti-HA (1:5000, Santa Cruz, F7 or 1:1000, 16B12, Covance), anti-Ubr3 (1:5000), anti-actin (1:5000, C4, MP Biomedicals), anti-α-tub (1:1000, Cell Signaling). The intensities of the bands in Fig 5B were quantified using image J software.

### In vitro ubiquitination assay

In vitro auto-ubiquitination assays were performed as described previously [119] with modifications. In brief, S2 cells were first transiently transfected with GFP, UBR-GFP or Ubr3-GFP. S2 cell cultures were collected 48 hours post-transfection and lysed on ice for 45 minutes under stringent conditions to minimize interactions with other proteins, using 100 μl RIPA buffer (150 mM NaCl, 1.0% NP-40, 0.5% sodium deoxycholate, 0.1% SDS, 50 mM Tris, pH 8.0) containing 1x Complete protease inhibitors cocktail (Roche) for every 10^6^ cells seeded. The lysates were then added to 30 μl bed volume of Chromotek GFP Trap beads (Allele Biotechnology), previously equilibrated with RIPA buffer, and incubated by rocking at 4°C for 3 hours. After washing with RIPA buffer, 20% of the beads were retained for assessing the expression of GFP protein by Western blot analysis using anti-GFP (1:1000, Zymed). The remainder of the lysates were equilibrated by rinsing twice in 1x Ubiquitination Reaction buffer (50 mM Tris-HCl, pH 7.4, 5 mM MgCl_2_, 50 mM NaCl, 1 mM dithiothreitol DTT, 1x protease inhibitors cocktail). The ubiquitination reaction was assembled by adding rabbit UBE1 E1 (Boston Biochem Cat.#302) and human recombinant His_6_-hHR6A E2 (Boston Biochem Cat. E2-612) conjugating enzymes and FLAG-Ubiquitin (Sigma) on ice and incubated at 30°C for 30 minutes. The reactions were stopped by adding 1x Laemmli buffer, after which the samples were boiled for 10 minutes and analyzed by SDS-PAGE and Western blot using anti-FLAG M2 monoclonal antibody (1:1000, Sigma).

For Cos2 ubiquitination assays, S2 cell culture and RNAi were performed as described previously [114]. Transfections were carried out using Effectene transfection reagent (Qiagen). The immunoprecipitation and immunoblot analysis were performed using standard protocols. Myc-Cos2 was constructed by fusion of 5xMyc tag to the N-terminus of Cos2 coding sequence. HA-Ub and Ub mutants have been described [34]. The HA::UbK48 only has mutations at all of the lysine residues with the exception of K48. GFP^RNAi^ has been described. Ubr3 dsRNA was synthesized against nucleotides 652–1191. The following antibodies were used: mouse anti-Myc (1:5000, 9E10, Santa Cruz), anti-GFP (1:1000, Millipore), anti-HA (1:5000, F7, Santa Cruz), and anti-β-tubulin (1:2000, E7, DSHB).

## Supporting Information

S1 Fig Hh signaling is defective in the *ubr3* complementation group. (A) *ubr3*^*A*^ mutant clones (labeled by dashed lines) crossing the morphogenetic furrow exhibit delayed differentiation of photoreceptors (arrow), revealed by expression of Senseless (cyan) and Elav (red). (B) *ubr3*^*A*^ mutant clones (labeled by dashed lines) exhibit loss of Ci^155^ staining (red) in the morphogenetic furrow and delay of R8 photoreceptor differentiation (arrow), visualized by Senseless expression (cyan). (C) Over-expression of p35 in *ubr3*^*B*^ mutant clones (labeled by dashed lines) does not rescue the loss of Ci^155^ (red) in the morphogenetic furrow (arrow). (D-E) TUNEL assays (red) were performed with eye discs bearing *ubr3*^*B/B*^ mutant clones (green in D) or *ubr3*^*B/B*^ *act>p35* clones (green in E). (F) The *ubr3* complementation group maps to *CG42593*. Both *ubr3*^*A*^ and *ubr3*^*B*^ alleles fail to complement *Df(1)BSC622* and a *P*-element insertion *P{lacW}CG42593*^*G0307a*^. A genomic rescue construct (symbolized by the green box) fully rescues the lethality of hemizygous *ubr3* mutants. (G) Over-expression of Ubr3 by *actin-Gal4* in *ubr3*^*B*^ mutant clones (outlined by dashed lines) fully restores the expression of Ci^155^ (red) in the morphogenetic furrow (arrow). (H) Structure of the genomic locus of *ubr3* gene. *ubr3* genomic rescue sequence is indicated in green box.(I) Identity (I) and similarity (S) of the three conserved domains between Ubr3 homologues from indicated species.
(TIF)

S2 Fig Subcellular localization of Ubr3. (A-G) S2 cells are co-stained with anti-Ubr3 antibody (red) and antibodies raised against different proteins associated with different organelles (green) and DAPI (blue). (H) Eye disc from 3^rd^ instar larvae in which *eyg-Gal4* drives expression of Ci^RNAi^ were stained with anti-Ci^155^ (red). Ci^155^ is reduced in the equator region of the morphogenetic furrow (arrow). (I) In situ hybridization experiments with an anti-*ubr3* probe were performed on eye discs from 3^rd^ instar larvae in which *eyg-Gal4* drove the expression of Ci^RNAi^. *ubr3* mRNA is reduced in the equator region of the morphogenetic furrow (arrow).
(TIF)

S3 Fig Cos2 and Cul1 are up-regulated in *ubr3* mutant cells. (A-C’) Co-immunolabeling of anti-Ptc (red) and anti-Senseless (cyan) (A-A’), anti-Fu (red) and anti-Senseless (cyan) (B-B’), anti-Cul1 (red) and anti-Senseless (cyan) (C-C’) in eye discs bearing *ubr3*^*B*^ mutant clones (labeled by dashed lines) from 3^rd^ instar larvae shows up-regulated Cul1 (red) in *ubr3*^*B*^ mutant cells. (D-D’) High magnification of the boundary region of *eyg-Gal4* driven expression of Cos2 in the eye disc. A solid line shows the boundary of the loss of Ci^155^ expression (shown in red). Cos2 levels are indicated by Cos2 labeling (shown in green). Arrows mark regions where Cos2 is expressed at low level (green) but is sufficient to inhibit Ci^155^ expression (red). (E-E’) Eye disc with *ubr3*^*B/B*^ clones that express Cos2 RNAi (shown in green) was labeled with anti-Sens (cyan). Arrows show the suppression of delayed differentiation of photoreceptor cells.
(TIF)

S4 Fig *ubr3*^*b1250*^ and *ubr3*^*b1251*^ mutants produce truncated, non-functional Ubr3 proteins. (A, B) DNA sequences show mutations found in *ubr3*^*b1250*^ and *ubr3*^*b1251*^ mutants. *ubr3*^*b1250*^ mutant carries a 28bp deletion around the CRISPR targeted region b1250. *ubr3*^*b1251*^ carries a 4bp insertion within the CRIPSR targeted region b1251. (C) Protein sequences of truncated Ubr3 proteins produced in *ubr3*^*b1250*^ and *ubr3*^*b1251*^ mutants.
(TIF)

S5 Fig *ubr3* is required for Hedgehog (Hh) signaling in zebrafish. (A, C) DIC images show lateral views of posterior trunk regions at 24-hours-post-fertilization. Anterior to the left, dorsal up. The angles of V-shaped somites were shown by the red line. Scale bars: 50μm. (B, D) *islet2* in situ hybridization (ISH) of wild type and *ubr3*^*b1250/1251*^ mutant zebrafish. Lateral views of posterior trunk regions at 24-hours-post-fertilization are shown. (E) Average somite angle in wild-type siblings (wt sibs) and *ubr3*^*b1250/b1251*^ trans-heterozygous mutants. Wild type siblings have a typical V-shaped somite characterized by an average angle of 92°. In the *ubr3* trans-heterozygous mutants, the angles become more obtuse with an average of 119°. (F-I) ISH against *ubr3* at 24 and 28 hpf. Lateral views of the somites. (F-G) At 24 hpf (F), *ubr3* is expressed throughout the somites. By 28hpf (G), this expression is restricted to ventral regions of the somites (red bracket). (H) *ubr3* expression is lost in *smo* mutants at 24 hpf. (I) At 28 hpf, the *ubr3* expression domain is expanded dorsally when Hh signaling is upregulated by ectopic expression of dnPKA (green bracket). Bars: SEM. Five angles were measured per larva. Five heterozygous and seven *ubr3*^*b1250/b1251*^ larvae were analyzed, P<0.01.
(TIF)

S6 Fig Ubr3 regulates Cos2 in the wing discs. (A-A’) Immunolabeling of wild type eye disc with anti-Cos2 (red) and anti-Ci^155^ (green). Arrow in A’ shows moderate increase of Cos2 protein levels in the morphogenetic furrow. (B-B’) Wing disc from 3^rd^ larvae with *ubr3*^*B*^ mutant clones (green) was stained with anti-Ci^155^ (red) and anti-senseless (sens, cyan). (C-C’) Wing disc from 3^rd^ larvae with *ubr3*^*B*^ mutant clones (green) was stained with anti-Cos2 (red) and anti-senseless (sens, cyan). (D-D’) A wing disc from 3^rd^ larvae with *ptc*^*DN*^ expressing clones (green) was stained with anti-Ci^155^ (red) and anti-senseless (sens, cyan). (E-E’) Wing disc from 3^rd^ larvae with *ubr3*^*B*^ mutant clones expressing *ptc*^*DN*^ (green) was stained with anti-Ci^155^ (red) and anti-senseless (sens, cyan). (F, H-J) In situ hybridizations were performed on wing discs from 3^rd^ instar larvae with indicated genotypes using an anti-*ubr3* probe. (G) A wing disc from 3^rd^ larvae in which *eyg-Gal4* driven expression of GFP was labeled with anti-GFP (green in G, indicating *eyg-Gal4* expression region), anti-Sens (cyan in G, labeling wing margin) and anti-Ci^155^ (red in G). Arrows indicate elevated ubr3 transcription in *eyg-Gal4* expressing domain.
(TIF)

## References

[1] Arwert EN, Hoste E, Watt FM. Epithelial stem cells, wound healing and cancer. Nat Rev Cancer. 2012;12(3):170–80. Epub 2012/03/01. 10.1038/nrc3217 . 22362215

[2] Varjosalo M, Taipale J. Hedgehog: functions and mechanisms. Genes & development. 2008;22(18):2454–72. Epub 2008/09/17. 10.1101/gad.1693608 . 18794343

[3] Babcock DT, Shi S, Jo J, Shaw M, Gutstein HB, Galko MJ. Hedgehog signaling regulates nociceptive sensitization. Curr Biol. 2011;21(18):1525–33. Epub 2011/09/13. 10.1016/j.cub.2011.08.020 21906949 PMC3262399

[4] Heemskerk J, DiNardo S. Drosophila hedgehog acts as a morphogen in cellular patterning. Cell. 1994;76(3):449–60. . 8313468 10.1016/0092-8674(94)90110-4

[5] Hooper JE, Scott MP. Communicating with Hedgehogs. Nature reviews. 2005;6(4):306–17. . 15803137 10.1038/nrm1622

[6] Lum L, Beachy PA. The Hedgehog response network: sensors, switches, and routers. Science (New York, NY. 2004;304(5678):1755–9. . 15205520 10.1126/science.1098020

[7] Jiang J, Hui CC. Hedgehog signaling in development and cancer. Developmental cell. 2008;15(6):801–12. 10.1016/j.devcel.2008.11.010 19081070 PMC6443374

[8] Pasca di Magliano M, Hebrok M. Hedgehog signalling in cancer formation and maintenance. Nat Rev Cancer. 2003;3(12):903–11. . 14737121 10.1038/nrc1229

[9] Taipale J, Cooper MK, Maiti T, Beachy PA. Patched acts catalytically to suppress the activity of Smoothened. Nature. 2002;418(6900):892–7. . 12192414 10.1038/nature00989

[10] Robbins DJ, Nybakken KE, Kobayashi R, Sisson JC, Bishop JM, Therond PP. Hedgehog elicits signal transduction by means of a large complex containing the kinesin-related protein costal2. Cell. 1997;90(2):225–34. Epub 1997/07/25. . 9244297 10.1016/s0092-8674(00)80331-1

[11] Sisson JC, Ho KS, Suyama K, Scott MP. Costal2, a novel kinesin-related protein in the Hedgehog signaling pathway. Cell. 1997;90(2):235–45. Epub 1997/07/25. . 9244298 10.1016/s0092-8674(00)80332-3

[12] Aikin RA, Ayers KL, Therond PP. The role of kinases in the Hedgehog signalling pathway. EMBO reports. 2008;9(4):330–6. 10.1038/embor.2008.38 18379584 PMC2288774

[13] Jiang J, Struhl G. Regulation of the Hedgehog and Wingless signalling pathways by the F-box/WD40-repeat protein Slimb. Nature. 1998;391(6666):493–6. Epub 1998/02/14. 10.1038/35154 . 9461217

[14] Jia J, Amanai K, Wang G, Tang J, Wang B, Jiang J. Shaggy/GSK3 antagonizes Hedgehog signalling by regulating Cubitus interruptus. Nature. 2002;416(6880):548–52. Epub 2002/03/26. 10.1038/nature733 . 11912487

[15] Jia J, Zhang L, Zhang Q, Tong C, Wang B, Hou F, et al Phosphorylation by double-time/CKIepsilon and CKIalpha targets cubitus interruptus for Slimb/beta-TRCP-mediated proteolytic processing. Developmental cell. 2005;9(6):819–30. Epub 2005/12/06. 10.1016/j.devcel.2005.10.006 . 16326393

[16] Zheng X, Mann RK, Sever N, Beachy PA. Genetic and biochemical definition of the Hedgehog receptor. Genes & development. 2010;24(1):57–71. . 20048000 10.1101/gad.1870310 PMC2802192

[17] Jia J, Tong C, Jiang J. Smoothened transduces Hedgehog signal by physically interacting with Costal2/Fused complex through its C-terminal tail. Genes & development. 2003;17(21):2709–20. Epub 2003/11/05. 10.1101/gad.1136603 14597665 PMC280620

[18] Lum L, Zhang C, Oh S, Mann RK, von Kessler DP, Taipale J, et al Hedgehog signal transduction via Smoothened association with a cytoplasmic complex scaffolded by the atypical kinesin, Costal-2. Molecular cell. 2003;12(5):1261–74. Epub 2003/11/26. . 14636583 10.1016/s1097-2765(03)00426-x

[19] Ogden SK, Ascano M Jr., Stegman MA, Suber LM, Hooper JE, Robbins DJ. Identification of a functional interaction between the transmembrane protein Smoothened and the kinesin-related protein Costal2. Curr Biol. 2003;13(22):1998–2003. Epub 2003/11/15. . 14614827 10.1016/j.cub.2003.10.004 PMC3711143

[20] Ruel L, Rodriguez R, Gallet A, Lavenant-Staccini L, Therond PP. Stability and association of Smoothened, Costal2 and Fused with Cubitus interruptus are regulated by Hedgehog. Nature cell biology. 2003;5(10):907–13. Epub 2003/10/03. 10.1038/ncb1052 . 14523402

[21] Zhang W, Zhao Y, Tong C, Wang G, Wang B, Jia J, et al Hedgehog-regulated Costal2-kinase complexes control phosphorylation and proteolytic processing of Cubitus interruptus. Developmental cell. 2005;8(2):267–78. . 15691767 10.1016/j.devcel.2005.01.001

[22] Ho KS, Suyama K, Fish M, Scott MP. Differential regulation of Hedgehog target gene transcription by Costal2 and Suppressor of Fused. Development (Cambridge, England). 2005;132(6):1401–12. . 15750186 10.1242/dev.01689

[23] Katoh Y, Katoh M. Characterization of KIF7 gene in silico. International journal of oncology. 2004;25(6):1881–6. . 15547730

[24] Katoh Y, Katoh M. KIF27 is one of orthologs for Drosophila Costal-2. International journal of oncology. 2004;25(6):1875–80. . 15547729

[25] Endoh-Yamagami S, Evangelista M, Wilson D, Wen X, Theunissen JW, Phamluong K, et al The mammalian Cos2 homolog Kif7 plays an essential role in modulating Hh signal transduction during development. Curr Biol. 2009;19(15):1320–6. 10.1016/j.cub.2009.06.046 19592253

[26] Liem KF Jr., He M, Ocbina PJ, Anderson KV. Mouse Kif7/Costal2 is a cilia-associated protein that regulates Sonic hedgehog signaling. Proceedings of the National Academy of Sciences of the United States of America. 2009;106(32):13377–82. Epub 2009/08/12. 10.1073/pnas.0906944106 19666503 PMC2726420

[27] Maurya AK, Ben J, Zhao Z, Lee RT, Niah W, Ng AS, et al Positive and negative regulation of Gli activity by Kif7 in the zebrafish embryo. PLoS genetics. 2013;9(12):e1003955 10.1371/journal.pgen.1003955 24339784 PMC3854788

[28] Putoux A, Thomas S, Coene KL, Davis EE, Alanay Y, Ogur G, et al KIF7 mutations cause fetal hydrolethalus and acrocallosal syndromes. Nature genetics. 2011;43(6):601–6. 10.1038/ng.826 21552264 PMC3674836

[29] Dafinger C, Liebau MC, Elsayed SM, Hellenbroich Y, Boltshauser E, Korenke GC, et al Mutations in KIF7 link Joubert syndrome with Sonic Hedgehog signaling and microtubule dynamics. The Journal of clinical investigation. 2011;121(7):2662–7. Epub 2011/06/03. 10.1172/JCI43639 21633164 PMC3223820

[30] Nybakken KE, Turck CW, Robbins DJ, Bishop JM. Hedgehog-stimulated phosphorylation of the kinesin-related protein Costal2 is mediated by the serine/threonine kinase fused. The Journal of biological chemistry. 2002;277(27):24638–47. . 11934882 10.1074/jbc.M110730200

[31] Ranieri N, Ruel L, Gallet A, Raisin S, Therond PP. Distinct phosphorylations on kinesin costal-2 mediate differential hedgehog signaling strength. Developmental cell. 2012;22(2):279–94. Epub 2012/02/07. 10.1016/j.devcel.2011.12.002 . 22306085

[32] Gulino A, Di Marcotullio L, Canettieri G, De Smaele E, Screpanti I. Hedgehog/Gli control by ubiquitination/acetylation interplay.: Elsevier Inc; 2012.

[33] Li S, Chen Y, Shi Q, Yue T, Wang B, Jiang J. Hedgehog-regulated ubiquitination controls smoothened trafficking and cell surface expression in Drosophila. PLoS biology. 2012;10(1):e1001239 10.1371/journal.pbio.1001239 22253574 PMC3254653

[34] Xia R, Jia H, Fan J, Liu Y, Jia J. USP8 promotes smoothened signaling by preventing its ubiquitination and changing its subcellular localization. PLoS biology. 2012;10(1):e1001238 Epub 2012/01/19. 10.1371/journal.pbio.1001238 22253573 PMC3254663

[35] Ordureau A, Munch C, Harper JW. Quantifying Ubiquitin Signaling. Molecular cell. 2015;58(4):660–76. 10.1016/j.molcel.2015.02.020 26000850 PMC4441763

[36] Metzger MB, Hristova VA, Weissman AM. HECT and RING finger families of E3 ubiquitin ligases at a glance. Journal of cell science. 2012;125(Pt 3):531–7. 10.1242/jcs.091777 22389392 PMC3381717

[37] Tasaki T, Zakrzewska A, Dudgeon DD, Jiang Y, Lazo JS, Kwon YT. The substrate recognition domains of the N-end rule pathway. The Journal of biological chemistry. 2009;284(3):1884–95. 10.1074/jbc.M803641200 19008229 PMC2615520

[38] Meisenberg C, Tait PS, Dianova II, Wright K, Edelmann MJ, Ternette N, et al Ubiquitin ligase UBR3 regulates cellular levels of the essential DNA repair protein APE1 and is required for genome stability. Nucleic acids research. 2012;40(2):701–11. Epub 2011/09/22. 10.1093/nar/gkr744 21933813 PMC3258136

[39] Tasaki T, Sohr R, Xia Z, Hellweg R, Hortnagl H, Varshavsky A, et al Biochemical and genetic studies of UBR3, a ubiquitin ligase with a function in olfactory and other sensory systems. The Journal of biological chemistry. 2007;282(25):18510–20. . 17462990 10.1074/jbc.M701894200

[40] Zanet J, Benrabah E, Li T, Pélissier-Monier A, Chanut-Delalande H, Ronsin B, et al Pri sORF peptides induce proteasome protein processing. Science (New York, NY. 2015. 10.1126/science.aac5677 26383956

[41] Huang Q, Tang X, Wang G, Fan Y, Ray L, Bergmann A, et al Ubr3 E3 ligase regulates apoptosis by controlling the activity of DIAP1 in Drosophila. Cell death and differentiation. 2014;21(12):1961–70. 10.1038/cdd.2014.115 25146930 PMC4227149

[42] Zhao C, Wang L, Ma X, Zhu W, Yao L, Cui Y, et al Cardiac Na 1.5 is modulated by ubiquitin protein ligase E3 component n-recognin UBR3 and 6. Journal of cellular and molecular medicine. 2015 . 26059563 10.1111/jcmm.12588 PMC4568919

[43] Yang S, Zhang H, Guo L, Zhao Y, Chen F. Reconstructing the coding and non-coding RNA regulatory networks of miRNAs and mRNAs in breast cancer. Gene. 2014;548(1):6–13. 10.1016/j.gene.2014.06.010 24979338

[44] Charng WL, Yamamoto S, Jaiswal M, Bayat V, Xiong B, Zhang K, et al Drosophila Tempura, a novel protein prenyltransferase alpha subunit, regulates notch signaling via Rab1 and Rab11. PLoS biology. 2014;12(1):e1001777 10.1371/journal.pbio.1001777 24492843 PMC3904817

[45] Haelterman NA, Jiang L, Li Y, Bayat V, Sandoval H, Ugur B, et al Large-scale identification of chemically induced mutations in Drosophila melanogaster. Genome research. 2014;24(10):1707–18. 10.1101/gr.174615.114 25258387 PMC4199363

[46] Xiong B, Bayat V, Jaiswal M, Zhang K, Sandoval H, Charng WL, et al Crag Is a GEF for Rab11 Required for Rhodopsin Trafficking and Maintenance of Adult Photoreceptor Cells. PLoS biology. 2012;10(12):e1001438 Epub 2012/12/12. 10.1371/journal.pbio.1001438 23226104 PMC3514319

[47] Yamamoto S, Charng WL, Rana NA, Kakuda S, Jaiswal M, Bayat V, et al A mutation in EGF repeat-8 of Notch discriminates between Serrate/Jagged and Delta family ligands. Science (New York, NY. 2012;338(6111):1229–32. Epub 2012/12/01. 10.1126/science.1228745 . 23197537 PMC3663443

[48] Yamamoto S, Jaiswal M, Charng WL, Gambin T, Karaca E, Mirzaa G, et al A drosophila genetic resource of mutants to study mechanisms underlying human genetic diseases. Cell. 2014;159(1):200–14. 10.1016/j.cell.2014.09.002 25259927 PMC4298142

[49] Frankfort BJ, Nolo R, Zhang Z, Bellen H, Mardon G. senseless repression of rough is required for R8 photoreceptor differentiation in the developing Drosophila eye. Neuron. 2001;32(3):403–14. Epub 2001/11/16. 11709152 10.1016/s0896-6273(01)00480-9 PMC3122332

[50] Nolo R, Abbott LA, Bellen HJ. Senseless, a Zn finger transcription factor, is necessary and sufficient for sensory organ development in Drosophila. Cell. 2000;102(3):349–62. . 10975525 10.1016/s0092-8674(00)00040-4

[51] Robinow S, White K. Characterization and spatial distribution of the ELAV protein during Drosophila melanogaster development. J Neurobiol. 1991;22(5):443–61. Epub 1991/07/01. 10.1002/neu.480220503 . 1716300

[52] Strutt DI, Mlodzik M. Hedgehog is an indirect regulator of morphogenetic furrow progression in the Drosophila eye disc. Development (Cambridge, England). 1997;124(17):3233–40. Epub 1997/10/06. . 9310318 10.1242/dev.124.17.3233

[53] Heberlein U, Wolff T, Rubin GM. The TGF beta homolog dpp and the segment polarity gene hedgehog are required for propagation of a morphogenetic wave in the Drosophila retina. Cell. 1993;75(5):913–26. Epub 1993/12/03. . 8252627 10.1016/0092-8674(93)90535-x

[54] Peter A, Schottler P, Werner M, Beinert N, Dowe G, Burkert P, et al Mapping and identification of essential gene functions on the X chromosome of Drosophila. EMBO reports. 2002;3(1):34–8. . 11751581 10.1093/embo-reports/kvf012 PMC1083931

[55] Tasaki T, Sriram SM, Park KS, Kwon YT. The N-end rule pathway. Annu Rev Biochem. 2012;81:261–89. Epub 2012/04/25. 10.1146/annurev-biochem-051710-093308 . 22524314 PMC3610525

[56] Johnson RL, Milenkovic L, Scott MP. In vivo functions of the patched protein: requirement of the C terminus for target gene inactivation but not Hedgehog sequestration. Molecular cell. 2000;6(2):467–78. . 10983992 10.1016/s1097-2765(00)00045-9

[57] Nagarkar-Jaiswal S, Lee PT, Campbell ME, Chen K, Anguiano-Zarate S, Gutierrez MC, et al A library of MiMICs allows tagging of genes and reversible, spatial and temporal knockdown of proteins in Drosophila. eLife. 2015;4 . 25824290 10.7554/eLife.05338 PMC4379497

[58] Ou CY, Lin YF, Chen YJ, Chien CT. Distinct protein degradation mechanisms mediated by Cul1 and Cul3 controlling Ci stability in Drosophila eye development. Genes & development. 2002;16(18):2403–14. Epub 2002/09/17. 10.1101/gad.1011402 12231629 PMC187440

[59] Deshaies RJ, Joazeiro CA. RING domain E3 ubiquitin ligases. Annu Rev Biochem. 2009;78:399–434. Epub 2009/06/06. 10.1146/annurev.biochem.78.101807.093809 . 19489725

[60] Koudijs MJ, den Broeder MJ, Groot E, van Eeden FJ. Genetic analysis of the two zebrafish patched homologues identifies novel roles for the hedgehog signaling pathway. BMC developmental biology. 2008;8:15 10.1186/1471-213X-8-15 18284698 PMC2275722

[61] Neumann CJ, Nuesslein-Volhard C. Patterning of the zebrafish retina by a wave of sonic hedgehog activity. Science (New York, NY. 2000;289(5487):2137–9. . 11000118 10.1126/science.289.5487.2137

[62] Wang X, Zhao Z, Muller J, Iyu A, Khng AJ, Guccione E, et al Targeted inactivation and identification of targets of the Gli2a transcription factor in the zebrafish. Biology open. 2013;2(11):1203–13. 10.1242/bio.20136262 24244857 PMC3828767

[63] Xu J, Srinivas BP, Tay SY, Mak A, Yu X, Lee SG, et al Genomewide expression profiling in the zebrafish embryo identifies target genes regulated by Hedgehog signaling during vertebrate development. Genetics. 2006;174(2):735–52. . 16888327 10.1534/genetics.106.061523 PMC1602081

[64] Lewis KE, Currie PD, Roy S, Schauerte H, Haffter P, Ingham PW. Control of muscle cell-type specification in the zebrafish embryo by Hedgehog signalling. Developmental biology. 1999;216(2):469–80. . 10642786 10.1006/dbio.1999.9519

[65] Schauerte HE, van Eeden FJ, Fricke C, Odenthal J, Strahle U, Haffter P. Sonic hedgehog is not required for the induction of medial floor plate cells in the zebrafish. Development (Cambridge, England). 1998;125(15):2983–93. . 9655820 10.1242/dev.125.15.2983

[66] van Eeden FJ, Granato M, Schach U, Brand M, Furutani-Seiki M, Haffter P, et al Mutations affecting somite formation and patterning in the zebrafish, Danio rerio. Development (Cambridge, England). 1996;123:153–64. . 9007237 10.1242/dev.123.1.153

[67] Varga ZM, Amores A, Lewis KE, Yan YL, Postlethwait JH, Eisen JS, et al Zebrafish smoothened functions in ventral neural tube specification and axon tract formation. Development (Cambridge, England). 2001;128(18):3497–509. . 11566855 10.1242/dev.128.18.3497

[68] Yeh CW, Kao SH, Cheng YC, Hsu LS. Knockdown of cyclin-dependent kinase 10 (cdk10) gene impairs neural progenitor survival via modulation of raf1a gene expression. The Journal of biological chemistry. 2013;288(39):27927–39. 10.1074/jbc.M112.420265 23902762 PMC3784707

[69] Nguyen VH, Trout J, Connors SA, Andermann P, Weinberg E, Mullins MC. Dorsal and intermediate neuronal cell types of the spinal cord are established by a BMP signaling pathway. Development (Cambridge, England). 2000;127(6):1209–20. . 10683174 10.1242/dev.127.6.1209

[70] Aanstad P, Santos N, Corbit KC, Scherz PJ, Trinh le A, Salvenmoser W, et al The extracellular domain of Smoothened regulates ciliary localization and is required for high-level Hh signaling. Curr Biol. 2009;19(12):1034–9. 10.1016/j.cub.2009.04.053 19464178 PMC2892286

[71] Hammond CL, Schulte-Merker S. Two populations of endochondral osteoblasts with differential sensitivity to Hedgehog signalling. Development (Cambridge, England). 2009;136(23):3991–4000. . 19906866 10.1242/dev.042150

[72] Hammond KL, Loynes HE, Folarin AA, Smith J, Whitfield TT. Hedgehog signalling is required for correct anteroposterior patterning of the zebrafish otic vesicle. Development (Cambridge, England). 2003;130(7):1403–17. . 12588855 10.1242/dev.00360

[73] Wolff C, Roy S, Ingham PW. Multiple muscle cell identities induced by distinct levels and timing of hedgehog activity in the zebrafish embryo. Curr Biol. 2003;13(14):1169–81. . 12867027 10.1016/s0960-9822(03)00461-5

[74] Wolff C, Roy S, Lewis KE, Schauerte H, Joerg-Rauch G, Kirn A, et al iguana encodes a novel zinc-finger protein with coiled-coil domains essential for Hedgehog signal transduction in the zebrafish embryo. Genes & development. 2004;18(13):1565–76. . 15198976 10.1101/gad.296004 PMC443519

[75] Hammerschmidt M, Bitgood MJ, McMahon AP. Protein kinase A is a common negative regulator of Hedgehog signaling in the vertebrate embryo. Genes & development. 1996;10(6):647–58. . 8598293 10.1101/gad.10.6.647

[76] Kinto N, Iwamoto M, Enomoto-Iwamoto M, Noji S, Ohuchi H, Yoshioka H, et al Fibroblasts expressing Sonic hedgehog induce osteoblast differentiation and ectopic bone formation. FEBS letters. 1997;404(2–3):319–23. . 9119087 10.1016/s0014-5793(97)00014-8

[77] Sinha S, Chen JK. Purmorphamine activates the Hedgehog pathway by targeting Smoothened. Nat Chem Biol. 2006;2(1):29–30. . 16408088 10.1038/nchembio753

[78] Cheung HO, Zhang X, Ribeiro A, Mo R, Makino S, Puviindran V, et al The kinesin protein Kif7 is a critical regulator of Gli transcription factors in mammalian hedgehog signaling. Science signaling. 2009;2(76):ra29 Epub 2009/06/25. 10.1126/scisignal.2000405 . 19549984

[79] Farzan SF, Ascano M Jr., Ogden SK, Sanial M, Brigui A, Plessis A, et al Costal2 functions as a kinesin-like protein in the hedgehog signal transduction pathway. Curr Biol. 2008;18(16):1215–20. Epub 2008/08/12. 10.1016/j.cub.2008.07.026 18691888 PMC2774813

[80] Wang G, Jiang J. Multiple Cos2/Ci interactions regulate Ci subcellular localization through microtubule dependent and independent mechanisms. Developmental biology. 2004;268(2):493–505. . 15063184 10.1016/j.ydbio.2004.01.008

[81] Zhu AJ, Zheng L, Suyama K, Scott MP. Altered localization of Drosophila Smoothened protein activates Hedgehog signal transduction. Genes & development. 2003;17(10):1240–52. Epub 2003/05/06. 10.1101/gad.1080803 12730121 PMC196058

[82] Huangfu D, Anderson KV. Signaling from Smo to Ci/Gli: conservation and divergence of Hedgehog pathways from Drosophila to vertebrates. Development (Cambridge, England). 2006;133(1):3–14. . 16339192 10.1242/dev.02169

[83] Chen MH, Gao N, Kawakami T, Chuang PT. Mice deficient in the fused homolog do not exhibit phenotypes indicative of perturbed hedgehog signaling during embryonic development. Molecular and cellular biology. 2005;25(16):7042–53. . 16055716 10.1128/MCB.25.16.7042-7053.2005 PMC1190231

[84] Merchant M, Evangelista M, Luoh SM, Frantz GD, Chalasani S, Carano RA, et al Loss of the serine/threonine kinase fused results in postnatal growth defects and lethality due to progressive hydrocephalus. Molecular and cellular biology. 2005;25(16):7054–68. . 16055717 10.1128/MCB.25.16.7054-7068.2005 PMC1190232

[85] Liu YC, Couzens AL, Deshwar AR, LD BM-C, Zhang X, Puviindran V, et al The PPFIA1-PP2A protein complex promotes trafficking of Kif7 to the ciliary tip and Hedgehog signaling. Science signaling. 2014;7(355):ra117 10.1126/scisignal.2005608 25492966

[86] Kalderon D. Similarities between the Hedgehog and Wnt signaling pathways. Trends in cell biology. 2002;12(11):523–31. . 12446114 10.1016/s0962-8924(02)02388-7

[87] Song X, Wang S, Li L. New insights into the regulation of Axin function in canonical Wnt signaling pathway. Protein & cell. 2014;5(3):186–93. . 24474204 10.1007/s13238-014-0019-2 PMC3967064

[88] Callow MG, Tran H, Phu L, Lau T, Lee J, Sandoval WN, et al Ubiquitin ligase RNF146 regulates tankyrase and Axin to promote Wnt signaling. PloS one. 2011;6(7):e22595 10.1371/journal.pone.0022595 21799911 PMC3143158

[89] DaRosa PA, Wang Z, Jiang X, Pruneda JN, Cong F, Klevit RE, et al Allosteric activation of the RNF146 ubiquitin ligase by a poly(ADP-ribosyl)ation signal. Nature. 2015;517(7533):223–6. 10.1038/nature13826 25327252 PMC4289021

[90] Zhang Y, Liu S, Mickanin C, Feng Y, Charlat O, Michaud GA, et al RNF146 is a poly(ADP-ribose)-directed E3 ligase that regulates axin degradation and Wnt signalling. Nature cell biology. 2011;13(5):623–9. 10.1038/ncb2222 21478859

[91] Lee T, Luo L. Mosaic analysis with a repressible cell marker for studies of gene function in neuronal morphogenesis. Neuron. 1999;22(3):451–61. . 10197526 10.1016/s0896-6273(00)80701-1

[92] Venken KJ, He Y, Hoskins RA, Bellen HJ. P[acman]: a BAC transgenic platform for targeted insertion of large DNA fragments in D. melanogaster. Science (New York, NY. 2006;314(5806):1747–51. Epub 2006/12/02. 10.1126/science.1134426 . 17138868

[93] Blackman RK, Sanicola M, Raftery LA, Gillevet T, Gelbart WM. An extensive 3' cis-regulatory region directs the imaginal disk expression of decapentaplegic, a member of the TGF-beta family in Drosophila. Development (Cambridge, England). 1991;111(3):657–66. Epub 1991/03/01. . 1908769 10.1242/dev.111.3.657

[94] Cook RK, Christensen SJ, Deal JA, Coburn RA, Deal ME, Gresens JM, et al The generation of chromosomal deletions to provide extensive coverage and subdivision of the Drosophila melanogaster genome. Genome biology. 2012;13(3):R21 10.1186/gb-2012-13-3-r21 22445104 PMC3439972

[95] Andrews HK, Giagtzoglou N, Yamamoto S, Schulze KL, Bellen HJ. Sequoia regulates cell fate decisions in the external sensory organs of adult Drosophila. EMBO reports. 2009;10(6):636–41. Epub 2009/05/16. 10.1038/embor.2009.66 19444309 PMC2711842

[96] Yao JG, Sun YH. Eyg and Ey Pax proteins act by distinct transcriptional mechanisms in Drosophila development. Embo J. 2005;24(14):2602–12. . 15973436 10.1038/sj.emboj.7600725 PMC1176454

[97] Dietzl G, Chen D, Schnorrer F, Su KC, Barinova Y, Fellner M, et al A genome-wide transgenic RNAi library for conditional gene inactivation in Drosophila. Nature. 2007;448(7150):151–6. Epub 2007/07/13. 10.1038/nature05954 . 17625558

[98] Ni JQ, Liu LP, Binari R, Hardy R, Shim HS, Cavallaro A, et al A Drosophila resource of transgenic RNAi lines for neurogenetics. Genetics. 2009;182(4):1089–100. 10.1534/genetics.109.103630 19487563 PMC2728850

[99] Westerfield M. The Zebrafish Book: A Guide for the Laboratory Use of Zebrafish (Danio rerio): Eugene, University of Oregon Press; 2007.

[100] Kimmel CB, Ballard WW, Kimmel SR, Ullmann B, Schilling TF. Stages of embryonic development of the zebrafish. Dev Dyn. 1995;203(3):253–310. . 8589427 10.1002/aja.1002030302

[101] Hwang WY, Fu Y, Reyon D, Maeder ML, Tsai SQ, Sander JD, et al Efficient genome editing in zebrafish using a CRISPR-Cas system. Nature biotechnology. 2013;31(3):227–9. 10.1038/nbt.2501 23360964 PMC3686313

[102] Jao LE, Wente SR, Chen W. Efficient multiplex biallelic zebrafish genome editing using a CRISPR nuclease system. Proceedings of the National Academy of Sciences of the United States of America. 2013;110(34):13904–9. 10.1073/pnas.1308335110 23918387 PMC3752207

[103] Sander JD, Maeder ML, Reyon D, Voytas DF, Joung JK, Dobbs D. ZiFiT (Zinc Finger Targeter): an updated zinc finger engineering tool. Nucleic acids research. 2010;38(Web Server issue):W462–8. 10.1093/nar/gkq319 20435679 PMC2896148

[104] Sander JD, Zaback P, Joung JK, Voytas DF, Dobbs D. Zinc Finger Targeter (ZiFiT): an engineered zinc finger/target site design tool. Nucleic acids research. 2007;35(Web Server issue):W599–605. . 17526515 10.1093/nar/gkm349 PMC1933188

[105] Motzny CK, Holmgren R. The Drosophila cubitus interruptus protein and its role in the wingless and hedgehog signal transduction pathways. Mech Dev. 1995;52(1):137–50. Epub 1995/07/01. . 7577671 10.1016/0925-4773(95)00397-j

[106] Capdevila J, Estrada MP, Sanchez-Herrero E, Guerrero I. The Drosophila segment polarity gene patched interacts with decapentaplegic in wing development. EMBO J. 1994;13(1):71–82. Epub 1994/01/01. 8306973 10.1002/j.1460-2075.1994.tb06236.x PMC394780

[107] Wu JT, Lin HC, Hu YC, Chien CT. Neddylation and deneddylation regulate Cul1 and Cul3 protein accumulation. Nature cell biology. 2005;7(10):1014–20. Epub 2005/08/30. 10.1038/ncb1301 . 16127432

[108] Chinchore Y, Mitra A, Dolph PJ. Accumulation of rhodopsin in late endosomes triggers photoreceptor cell degeneration. PLoS genetics. 2009;5(2):e1000377 10.1371/journal.pgen.1000377 19214218 PMC2633617

[109] Khodosh R, Augsburger A, Schwarz TL, Garrity PA. Bchs, a BEACH domain protein, antagonizes Rab11 in synapse morphogenesis and other developmental events. Development (Cambridge, England). 2006;133(23):4655–65. . 17079274 10.1242/dev.02650

[110] Baqri RM, Turner BA, Rheuben MB, Hammond BD, Kaguni LS, Miller KE. Disruption of mitochondrial DNA replication in Drosophila increases mitochondrial fast axonal transport in vivo. PloS one. 2009;4(11):e7874 10.1371/journal.pone.0007874 19924234 PMC2773408

[111] Blanco-Sanchez B, Clement A, Fierro J Jr., Washbourne P, Westerfield M. Complexes of Usher proteins preassemble at the endoplasmic reticulum and are required for trafficking and ER homeostasis. Disease models & mechanisms. 2014;7(5):547–59. . 24626987 10.1242/dmm.014068 PMC4007406

[112] dos Santos G, Schroeder AJ, Goodman JL, Strelets VB, Crosby MA, Thurmond J, et al FlyBase: introduction of the Drosophila melanogaster Release 6 reference genome assembly and large-scale migration of genome annotations. Nucleic acids research. 2014;43(Database issue):D690–7. 10.1093/nar/gku1099 25398896 PMC4383921

[113] Bischof J, Maeda RK, Hediger M, Karch F, Basler K. An optimized transgenesis system for Drosophila using germ-line-specific phiC31 integrases. Proceedings of the National Academy of Sciences of the United States of America. 2007;104(9):3312–7. . 17360644 10.1073/pnas.0611511104 PMC1805588

[114] Liu Y, Cao X, Jiang J, Jia J. Fused-Costal2 protein complex regulates Hedgehog-induced Smo phosphorylation and cell-surface accumulation. Genes & development. 2007;21(15):1949–63. Epub 2007/08/03. 10.1101/gad.1557407 17671093 PMC1935032

[115] Wei W. Regulatory signaling in breast cancer stem cells (doctoral dissertation). Baylor College of Medicine, Houston, TX 2014.

[116] Reya T, Duncan AW, Ailles L, Domen J, Scherer DC, Willert K, et al A role for Wnt signalling in self-renewal of haematopoietic stem cells. Nature. 2003;423(6938):409–14. . 12717450 10.1038/nature01593

[117] Sturtevant MA, Roark M, Bier E. The Drosophila rhomboid gene mediates the localized formation of wing veins and interacts genetically with components of the EGF-R signaling pathway. Genes & development. 1993;7(6):961–73. . 8504935 10.1101/gad.7.6.961

[118] Thisse B, Heyer V, Lux A, Alunni V, Degrave A, Seiliez I, et al Spatial and temporal expression of the zebrafish genome by large-scale in situ hybridization screening. Methods in cell biology. 2004;77:505–19. . 15602929 10.1016/s0091-679x(04)77027-2

[119] Furukawa M, Andrews PS, Xiong Y. Assays for RING family ubiquitin ligases. Methods Mol Biol. 2005;301:37–46. Epub 2005/05/27. 10.1385/1-59259-895-1:037 . 15917624

