# Supplementary figures and images for: Ubr3, a Novel Modulator of Hh Signaling Affects the Degradation of Costal-2 and Kif7 through Poly-ubiquitination
Source: PLoS Genet. 2016 May 19;12(5):e1006054. doi: 10.1371/journal.pgen.1006054 (PMC4873228; doi:10.1371/journal.pgen.1006054)

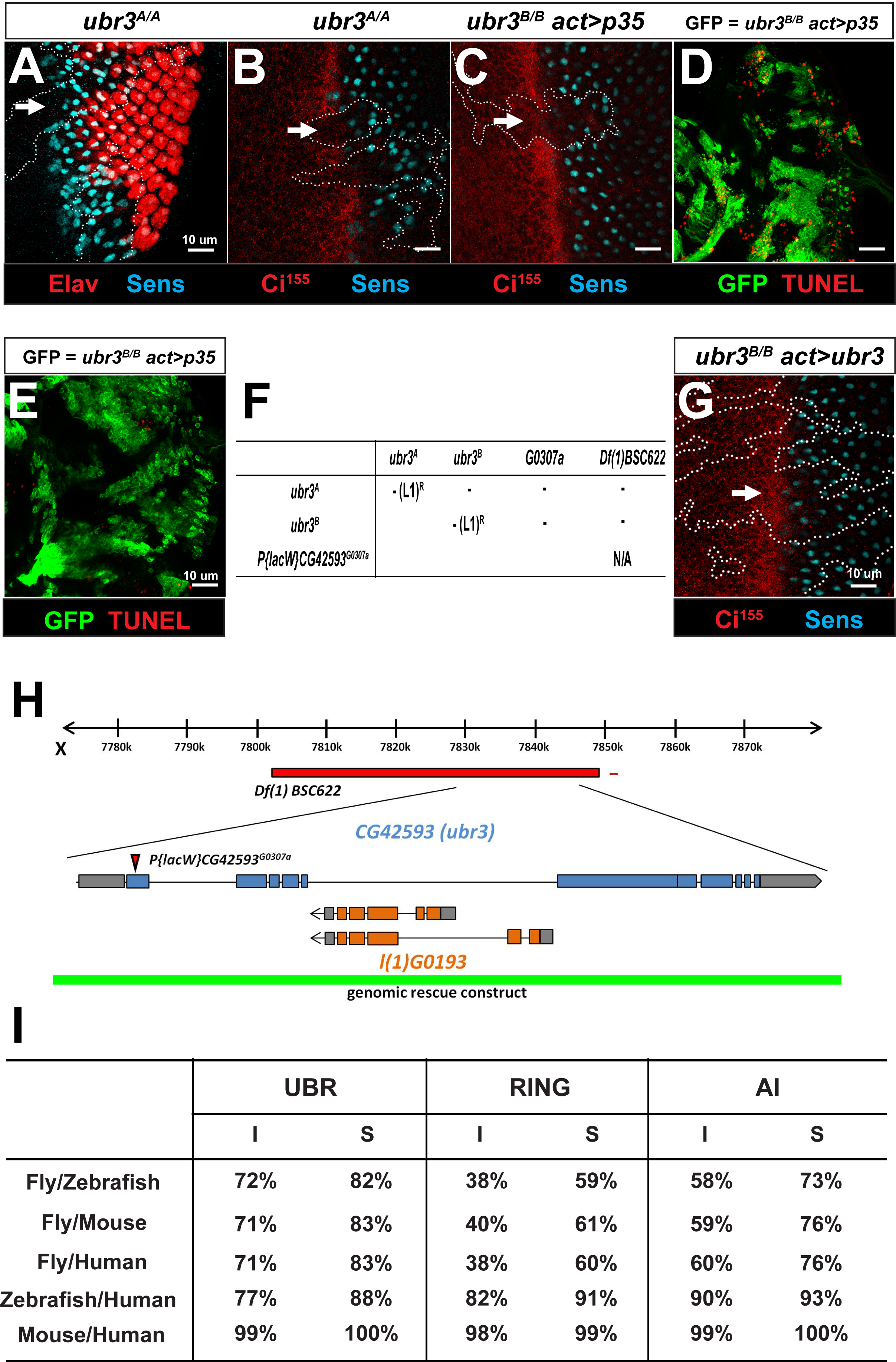

Supplement: S1 Fig — (A) ubr3A mutant clones (labeled by dashed lines) crossing the morphogenetic furrow exhibit delayed differentiation of photoreceptors (arrow), revealed by expression of Senseless (cyan) and Elav (red). (B) ubr3A mutant clones (labeled by dashed lines) exhibit loss of Ci155 staining (red) in the morphogenetic furrow and delay of R8 photoreceptor differentiation (arrow), visualized by Senseless expression (cyan). (C) Over-expression of p35 in ubr3B mutant clones (labeled by dashed lines) does not rescue the loss of Ci155 (red) in the morphogenetic furrow (arrow). (D-E) TUNEL assays (red) were performed with eye discs bearing ubr3B/B mutant clones (green in D) or ubr3B/B act>p35 clones (green in E). (F) The ubr3 complementation group maps to CG42593. Both ubr3A and ubr3B alleles fail to complement Df(1)BSC622 and a P-element insertion P{lacW}CG42593G0307a. A genomic rescue construct (symbolized by the green box) fully rescues the lethality of hemizygous ubr3 mutants. (G) Over-expression of Ubr3 by actin-Gal4 in ubr3B mutant clones (outlined by dashed lines) fully restores the expression of Ci155 (red) in the morphogenetic furrow (arrow). (H) Structure of the genomic locus of ubr3 gene. ubr3 genomic rescue sequence is indicated in green box.(I) Identity (I) and similarity (S) of the three conserved domains between Ubr3 homologues from indicated species. (TIF) [file pgen.1006054.s001.tif]

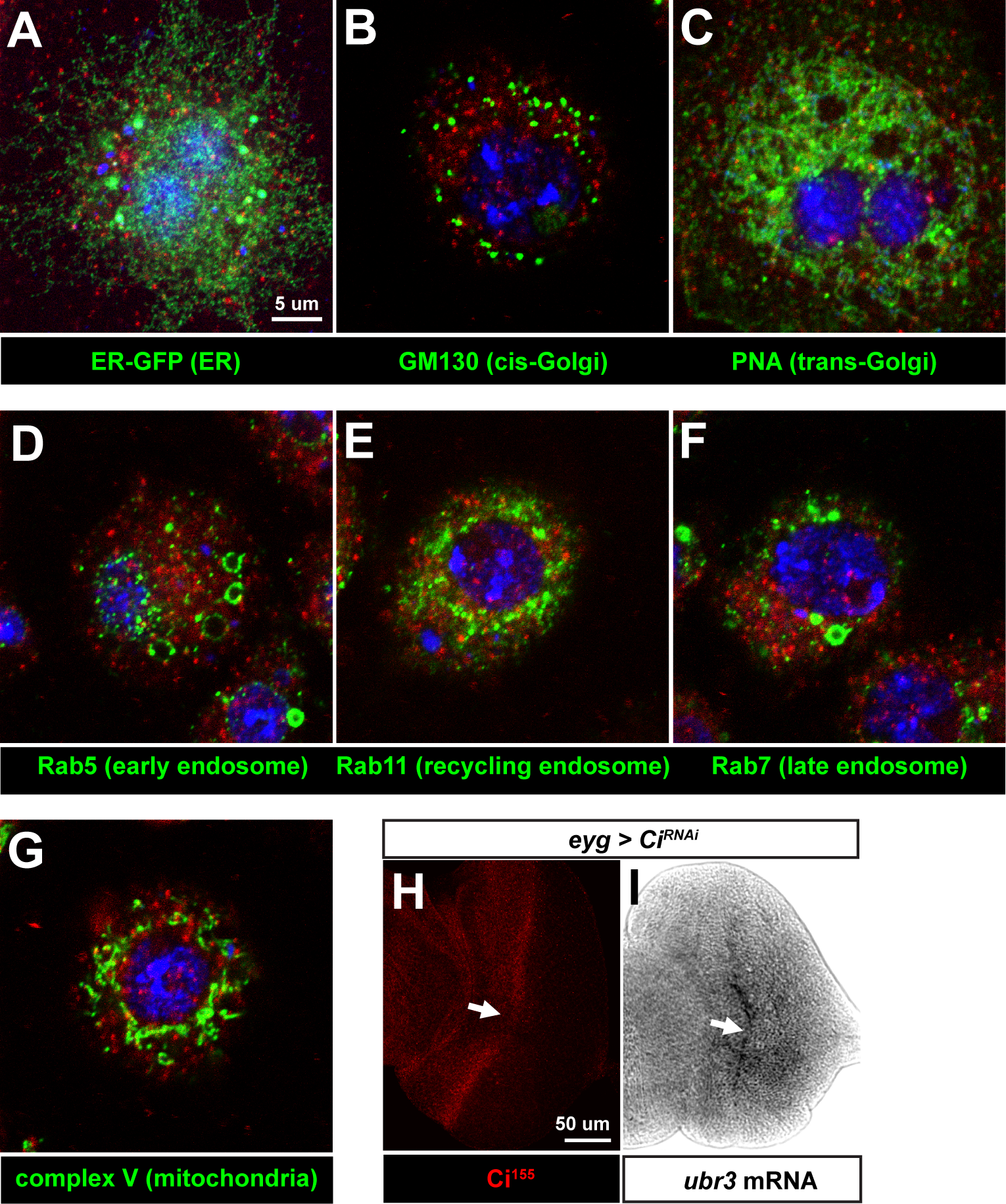

Supplement: S2 Fig — (A-G) S2 cells are co-stained with anti-Ubr3 antibody (red) and antibodies raised against different proteins associated with different organelles (green) and DAPI (blue). (H) Eye disc from 3rd instar larvae in which eyg-Gal4 drives expression of CiRNAi were stained with anti-Ci155 (red). Ci155 is reduced in the equator region of the morphogenetic furrow (arrow). (I) In situ hybridization experiments with an anti-ubr3 probe were performed on eye discs from 3rd instar larvae in which eyg-Gal4 drove the expression of CiRNAi. ubr3 mRNA is reduced in the equator region of the morphogenetic furrow (arrow). (TIF) [file pgen.1006054.s002.tif]

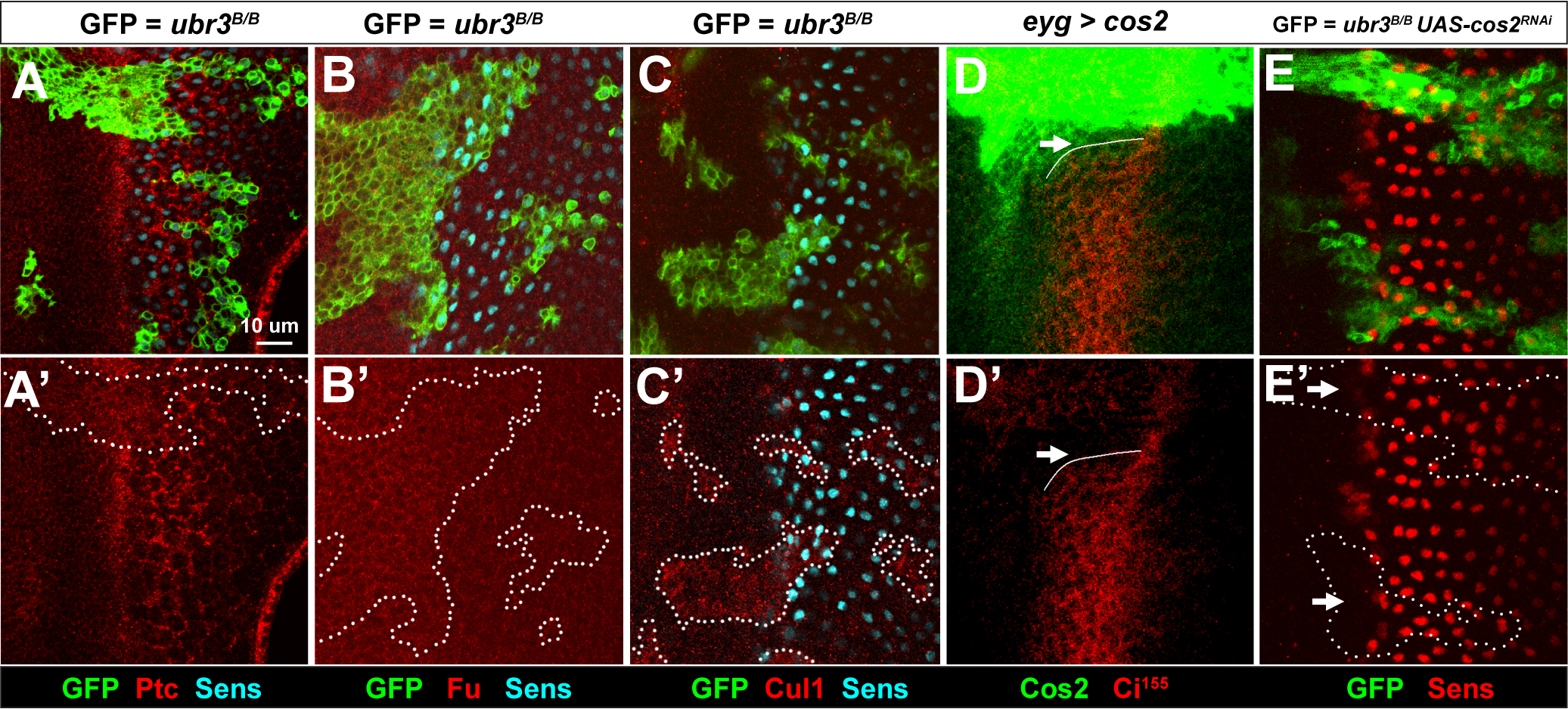

Supplement: S3 Fig — (A-C’) Co-immunolabeling of anti-Ptc (red) and anti-Senseless (cyan) (A-A’), anti-Fu (red) and anti-Senseless (cyan) (B-B’), anti-Cul1 (red) and anti-Senseless (cyan) (C-C’) in eye discs bearing ubr3B mutant clones (labeled by dashed lines) from 3rd instar larvae shows up-regulated Cul1 (red) in ubr3B mutant cells. (D-D’) High magnification of the boundary region of eyg-Gal4 driven expression of Cos2 in the eye disc. A solid line shows the boundary of the loss of Ci155 expression (shown in red). Cos2 levels are indicated by Cos2 labeling (shown in green). Arrows mark regions where Cos2 is expressed at low level (green) but is sufficient to inhibit Ci155 expression (red). (E-E’) Eye disc with ubr3B/B clones that express Cos2 RNAi (shown in green) was labeled with anti-Sens (cyan). Arrows show the suppression of delayed differentiation of photoreceptor cells. (TIF) [file pgen.1006054.s003.tif]

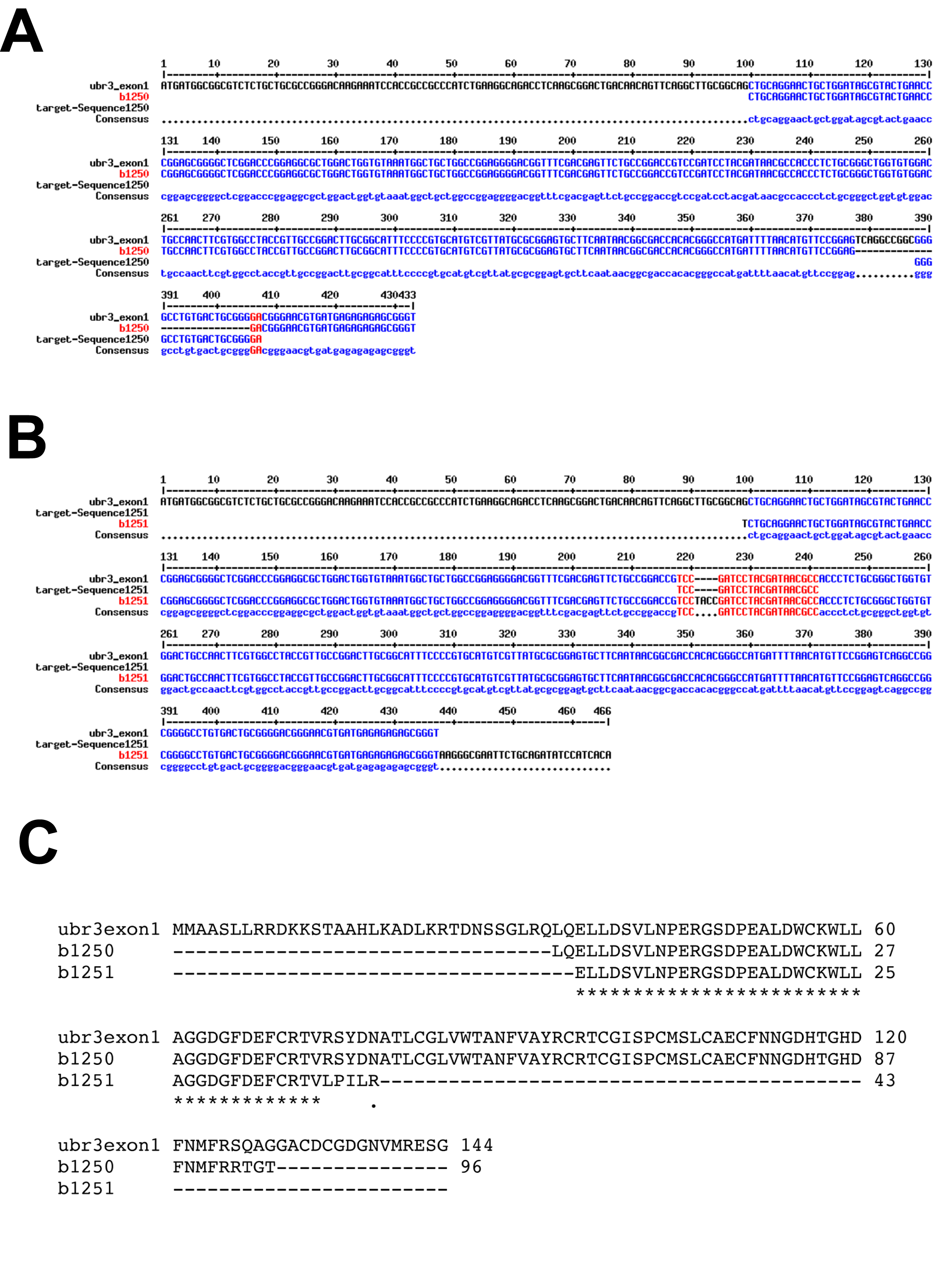

Supplement: S4 Fig — (A, B) DNA sequences show mutations found in ubr3b1250 and ubr3b1251 mutants. ubr3b1250 mutant carries a 28bp deletion around the CRISPR targeted region b1250. ubr3b1251 carries a 4bp insertion within the CRIPSR targeted region b1251. (C) Protein sequences of truncated Ubr3 proteins produced in ubr3b1250 and ubr3b1251 mutants. (TIF) [file pgen.1006054.s004.tif]

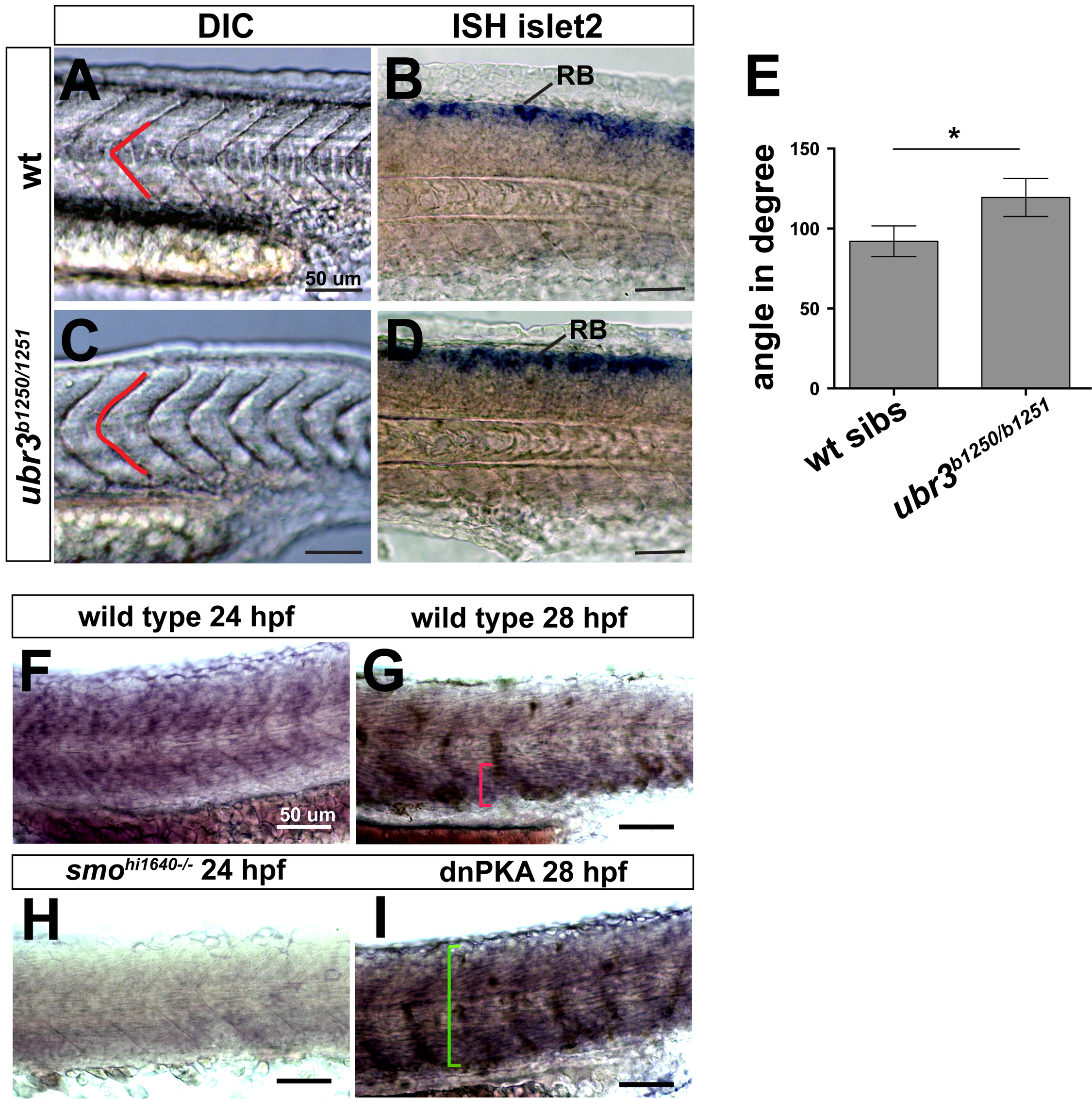

Supplement: S5 Fig — (A, C) DIC images show lateral views of posterior trunk regions at 24-hours-post-fertilization. Anterior to the left, dorsal up. The angles of V-shaped somites were shown by the red line. Scale bars: 50μm. (B, D) islet2 in situ hybridization (ISH) of wild type and ubr3b1250/1251 mutant zebrafish. Lateral views of posterior trunk regions at 24-hours-post-fertilization are shown. (E) Average somite angle in wild-type siblings (wt sibs) and ubr3b1250/b1251 trans-heterozygous mutants. Wild type siblings have a typical V-shaped somite characterized by an average angle of 92°. In the ubr3 trans-heterozygous mutants, the angles become more obtuse with an average of 119°. (F-I) ISH against ubr3 at 24 and 28 hpf. Lateral views of the somites. (F-G) At 24 hpf (F), ubr3 is expressed throughout the somites. By 28hpf (G), this expression is restricted to ventral regions of the somites (red bracket). (H) ubr3 expression is lost in smo mutants at 24 hpf. (I) At 28 hpf, the ubr3 expression domain is expanded dorsally when Hh signaling is upregulated by ectopic expression of dnPKA (green bracket). Bars: SEM. Five angles were measured per larva. Five heterozygous and seven ubr3b1250/b1251 larvae were analyzed, P<0.01. (TIF) [file pgen.1006054.s005.tif]

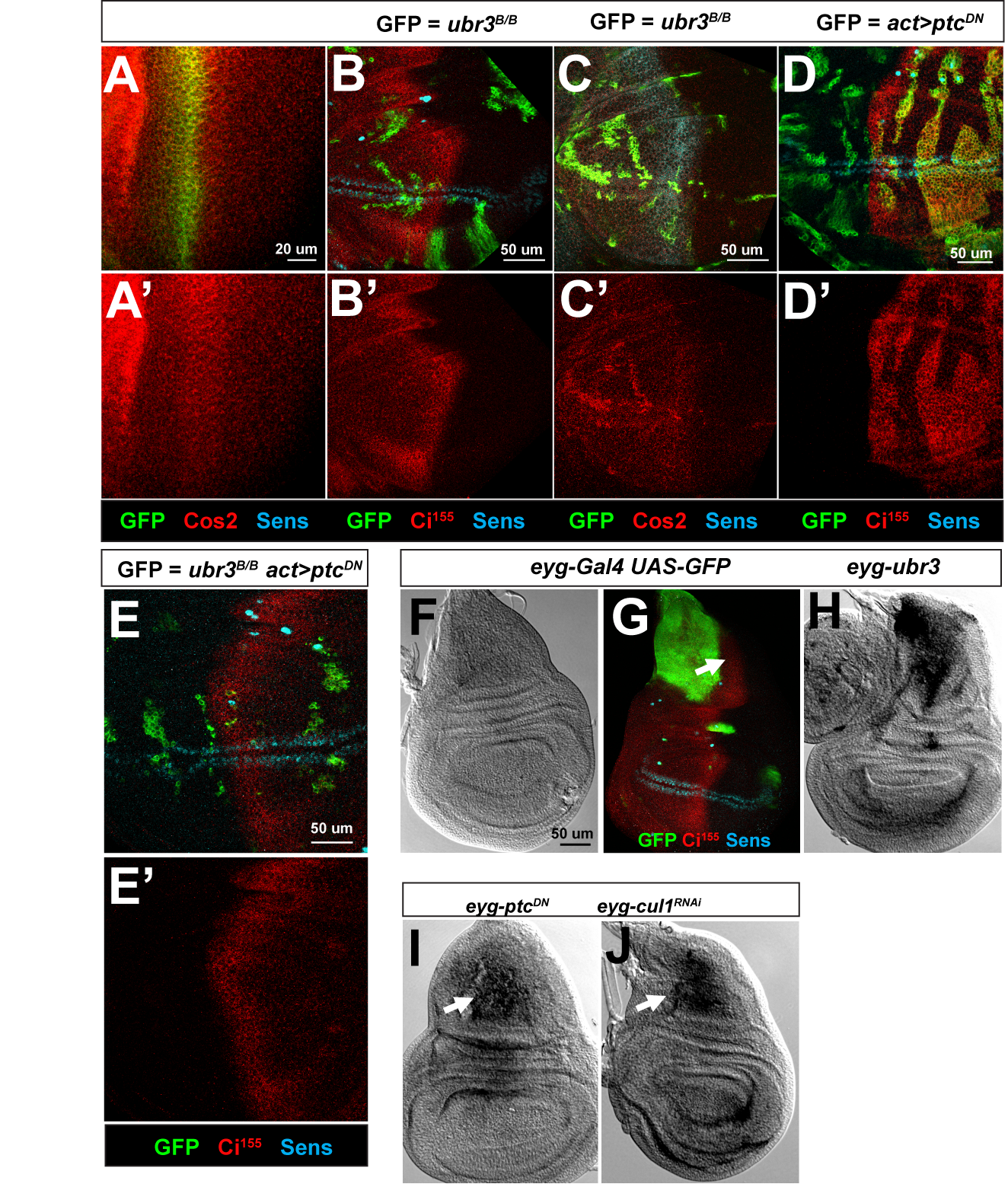

Supplement: S6 Fig — (A-A’) Immunolabeling of wild type eye disc with anti-Cos2 (red) and anti-Ci155 (green). Arrow in A’ shows moderate increase of Cos2 protein levels in the morphogenetic furrow. (B-B’) Wing disc from 3rd larvae with ubr3B mutant clones (green) was stained with anti-Ci155 (red) and anti-senseless (sens, cyan). (C-C’) Wing disc from 3rd larvae with ubr3B mutant clones (green) was stained with anti-Cos2 (red) and anti-senseless (sens, cyan). (D-D’) A wing disc from 3rd larvae with ptcDN expressing clones (green) was stained with anti-Ci155 (red) and anti-senseless (sens, cyan). (E-E’) Wing disc from 3rd larvae with ubr3B mutant clones expressing ptcDN (green) was stained with anti-Ci155 (red) and anti-senseless (sens, cyan). (F, H-J) In situ hybridizations were performed on wing discs from 3rd instar larvae with indicated genotypes using an anti-ubr3 probe. (G) A wing disc from 3rd larvae in which eyg-Gal4 driven expression of GFP was labeled with anti-GFP (green in G, indicating eyg-Gal4 expression region), anti-Sens (cyan in G, labeling wing margin) and anti-Ci155 (red in G). Arrows indicate elevated ubr3 transcription in eyg-Gal4 expressing domain. (TIF) [file pgen.1006054.s006.tif]
